# A Review of Advanced Multifunctional Magnetic Nanostructures for Cancer Diagnosis and Therapy Integrated into an Artificial Intelligence Approach

**DOI:** 10.3390/pharmaceutics15030868

**Published:** 2023-03-07

**Authors:** Bharath Govindan, Muhammad Ashraf Sabri, Abdul Hai, Fawzi Banat, Mohammad Abu Haija

**Affiliations:** 1 Department of Chemical Engineering, Khalifa University of Science and Technology, Abu Dhabi P.O. Box 127788, United Arab Emirates; 2 Department of Chemistry, Khalifa University of Science and Technology, Abu Dhabi P.O. Box 127788, United Arab Emirates; 3 Advanced Materials Chemistry Center (AMCC), Khalifa University of Science and Technology, Abu Dhabi P.O. Box 127788, United Arab Emirates

**Keywords:** magnetic nanostructures, smart magnetic nanoparticles, cancer diagnostics, cancer therapies, artificial neural network

## Abstract

The new era of nanomedicine offers significant opportunities for cancer diagnostics and treatment. Magnetic nanoplatforms could be highly effective tools for cancer diagnosis and treatment in the future. Due to their tunable morphologies and superior properties, multifunctional magnetic nanomaterials and their hybrid nanostructures can be designed as specific carriers of drugs, imaging agents, and magnetic theranostics. Multifunctional magnetic nanostructures are promising theranostic agents due to their ability to diagnose and combine therapies. This review provides a comprehensive overview of the development of advanced multifunctional magnetic nanostructures combining magnetic and optical properties, providing photoresponsive magnetic platforms for promising medical applications. Moreover, this review discusses various innovative developments using multifunctional magnetic nanostructures, including drug delivery, cancer treatment, tumor-specific ligands that deliver chemotherapeutics or hormonal agents, magnetic resonance imaging, and tissue engineering. Additionally, artificial intelligence (AI) can be used to optimize material properties in cancer diagnosis and treatment, based on predicted interactions with drugs, cell membranes, vasculature, biological fluid, and the immune system to enhance the effectiveness of therapeutic agents. Furthermore, this review provides an overview of AI approaches used to assess the practical utility of multifunctional magnetic nanostructures for cancer diagnosis and treatment. Finally, the review presents the current knowledge and perspectives on hybrid magnetic systems as cancer treatment tools with AI models.

## 1. Introduction

Cancer is a condition that causes uncontrollable growth of cells within the body. The characteristics of cancer include abnormal differentiation, proliferation, loss of control, infiltration, and metastatic spread [1]. The number of new cases of cancer increases every year, making it one of the world’s deadliest diseases. Cancers are generally classified as leukemia, lymphoma, sarcoma, melanoma, and carcinoma [2]. Lymphoma is a cancer of lymphocytes, whereas leukemia is a type of blood cancer. Sarcoma can appear in a variety of soft or connective tissues, such as bone, muscle, fat, blood vessels, or cartilage. Melanoma is another cancer type that affects and targets the skin pigment cells. The most common type of cancer is carcinoma, which can affect the pancreas, breasts, skin, lungs, or other organs [3]. Cancer is currently treated with surgery, chemotherapy, radiation therapy, targeted therapy, immunotherapy, stem cell or bone marrow transplant, and hormone therapy. Surgery is the most commonly used and basic method of resecting lesions [4]. A lymphadenectomy can improve the effectiveness of surgery, but incomplete resection still increases the risk of metastasizing cancer. To remove cancerous lesions, the use of chemotherapy involves the use of specific drugs, whereas radiotherapy involves the use of radiation. Targeted therapy delivers a drug directly to cancer cells through a variety of nanocarriers, which makes the treatment more precise and effective [5]. Currently, other cancer therapies are not mature enough to treat cancer accurately.

During the past few years, nanotechnology has been involved in chemotherapy, radiation therapy, and targeted therapy for the treatment of various types of cancer [6]. Nanomaterials are used in modern nanomedicine for developing early diagnostic, detection, and treatment methods [7,8]. Several factors affect the potential biomedical applications, including porosity, size, surface functional groups, electronic properties, zeta potential, and possible interactions [9,10,11]. Modern nanomedicine requires optimizing the design and physiochemical characteristics of nanohybrid nanostructures before tackling other significant issues. Especially, advances in hybrid magnetic nanostructures research have had a groundbreaking impact on biomedical applications [12]. Hybrid magnetic nanostructures (MHNs) can be used in magnetic separation, diagnostics, cancer drug delivery, in vivo imaging of cancer, and as contrast agents in MRIs [13,14]. A great deal of effort has been spent on developing ferromagnetic MHNs with controlled parameters [15,16,17,18]. MHNs are currently being developed and utilized in many clinical applications including cancer diagnosis and treatment. Moreover, cancer diagnosis and treatment can benefit greatly from the use of artificial neural networks (ANN), although this field is still in its infancy [19]. ANN algorithms can be used to optimize nanopharmaceuticals formulations for enhanced transport and targeting of nanomedicines through the prediction of interactions between MHNs nanocarriers, drugs, biological mediators, or cell membranes, as well as the estimation of drug encapsulation efficiency [20]. In addition, ANN can improve clinical outcomes while reducing toxicity, by improving the efficiency of drug delivery and design of the MHNs [21,22,23,24]. The purpose of this section is to provide an overview of the development and implementation of MHNs for cancer diagnosis and treatment using ANN approaches.

## 2. Magnetic Nanomaterials and Their Magnetic Hybrids Nanostructures (MHNs)

The advent and development of nanomedicine offer new avenues to improve conventional cancer therapies. Magnetic nanomaterials and hybrid nanostructures are set to hold a lot of interest in the future because of their physicochemical properties, adjustable size and shape, and ease of functionalization. In biomedical applications, iron oxide nanoparticles (IONPs), especially maghemite and magnetite oxides, ferrites are commonly used because of their ability to decompose in the body and release oxygen and iron [25]. They can be easily excreted from the body after degradation through oxygen transport and metabolic systems. Hence, understanding the physicochemical properties, such as size- and shape-dependent properties, composition, and functionality of magnetic nanoparticles is crucial when these materials are used in modern cancer diagnosis and therapy. An overview of different types of magnetic nanomaterials and surface functionalization strategies is provided in this section.

### 2.1. Morphological Effects of Magnetic Nanomaterials on Cancer Diagnosis and Treatment

When nanoparticles with a diameter of approximately 10 nm are synthesized, they exhibit superparamagnetic properties due to better dispersibility without a magnetic field [26,27]. Cancer therapy relies on the accumulation of these compounds at a target site in the presence of a magnetic field. The size, shape, and surface coating of magnetic nanoparticles can all play a role in their effectiveness for cancer applications such as drug delivery, imaging, hyperthermia, and theranostics [18,28]. The size of magnetic nanoparticles can influence their behavior in cancer therapy. Smaller nanoparticles (~10 nm) tend to be more stable and have a higher surface area-to-volume ratio, which can make them more effective for drug delivery and imaging. However, larger nanoparticles (~50 nm) may be more effective in hyperthermia treatment to kill cancer cells by utilizing heat [29]. The shape of magnetic nanoparticles can also influence their behavior in cancer therapy. For example, rod-shaped nanoparticles may be more effective at inducing hyperthermia than spherical nanoparticles. Magnetic nanoparticles (MNPs) with rod shapes have greater magnetic torque, more intense oscillation, and a greater area involved in the AMF, which results in a higher hyperthermia effect. Moreover, the demagnetization effect indirectly influenced the morphological features through the coercivity of the MNPs. MNPs with rod-shaped shapes had similar saturation magnetic inductions, but their coercivity was 110.42 Gs, which was twice as high as that of spheres (53.185 Gs) [30]. Rod-shaped MNPs consume more energy in vibration than spherical MNPs, i.e., mechanical movement consumes more energy [30,31]. Furthermore, the surface coating of magnetic nanoparticles can influence their stability, biocompatibility, and ability to target cancer cells. For example, nanoparticles coated with biomolecules, such as antibodies or peptides, may be more effective at targeting cancer cells [32].

On the other hand, smaller MNPs can more easily enter into the cancerous tissues and accumulate at the tumor site due to the enhanced permeability and retention (EPR) effect [32,33]. Larger MNPs may have a higher payload capacity but may have lesser diffusivity in the tumor tissue. In drug delivery, magnetic nanoparticles with smaller diameters may be able to target cancer cells and release their payloads, such as chemotherapy drugs or gene therapies, more effectively [34]. This can help to minimize the side effects of treatment and improve the overall effectiveness of the therapy. For example, magnetic nanoparticles can be used to deliver chemotherapy drugs to cancer cells or to deliver gene therapies to modify the expression of specific genes in cancer cells. Magnetic nanoparticles can be used for imaging cancer cells in vivo. Smaller magnetic nanoparticles tend to be more effective at producing high-contrast images of cancer cells and tissues, as they have a higher surface area-to-volume ratio and are more susceptible to the magnetic field [35,36,37]. In hyperthermia, larger magnetic nanoparticles may be more effective at inducing heat in cancer cells. This can be achieved by exposing the nanoparticles to an alternating magnetic field, which causes them to oscillate and generate heat. The heat generated by the nanoparticles can then be used to kill cancer cells while minimizing the impact on healthy cells [28]. The quality and effectiveness of MNPs mainly depend on the size and shape of nanoparticles in the final product. The size of the MNPs can be effectively controlled by suitable synthesis methods and reaction conditions. The most important parameters are solvent, pH surfactant, reaction temperature, pressure, residence time, salt source, and precursor. Park et al. reported a large-scale synthesis method for monodisperse nanocrystal synthesis within a size range of 5–22 nm using inexpensive metal salts as reactants in varying solvents [38]. Peng et al. reported the synthesis of self-assembled amorphous core-shell Fe–Fe_3_O_4_ nanoparticles within a controlled size-range of 2.5–3.5 nm [39].

Overall, the size of magnetic nanoparticles can play a role in their effectiveness for cancer therapy, depending on the specific application. Further research is needed to fully understand the optimal size of magnetic nanoparticles for different cancer therapy applications. Magnetic nanoparticles have been explored as a potential tool for cancer therapy due to their ability to be selectively delivered to cancer cells and then activated using an external magnetic field [40]. The shape of the magnetic nanoparticles can affect their behavior and performance in cancer therapy applications such as hyperthermia and targeted drug delivery. Several morphologies such as spherical, octahedrons, rods, plates, cubes, rings, hexagons, capsules, wires, tubes, and flower-shaped, depending on the reaction conditions, have been reported in the literature for MNPs suitable for different cancer treatment and therapy applications [40,41]. The shape of MNPs is a key factor in determining their effectiveness in cancer therapy. Research has shown that MNPs with different shapes can have different properties, such as magnetic moments, surface area, stability, binding affinity with certain drugs, and their ability to deliver a uniform distribution of drug payload [42]. These properties can influence the behavior of the MNPs in the body, as well as their ability to target and treat cancer cells. For example, rod-shaped MNPs may have a higher binding affinity for certain drugs, whereas sphere-shaped MNPs may have a more uniform distribution of drug payload [43]. Magnetic nanoparticles with spherical shapes penetrate tissues better than rods and wires and can reach cancer cells more easily. They may also be more easily activated using a magnetic field, as the longer shape allows for a stronger interaction with the field [44]. Additionally, rod-shaped MNPs may have a higher binding affinity for certain drugs, whereas sphere-shaped MNPs may have a more uniform distribution of drug payload. On the other hand, spherical particles may be more stable and easier to synthesize and may also have a lower toxicity profile [45]. MNPs that are spherical or ellipsoidal tend to have higher stability and lower toxicity compared to MNPs with more complex shapes [46].

This makes them more suitable for use in cancer therapy, as they are less likely to cause side effects. On the other hand, MNPs with more complex shapes, such as rod- or wire-shaped MNPs, tend to have a higher surface area and a stronger magnetic moment. Nanocube morphologies can have a better response for guided chemo-photothermal therapy [47]. This can make them more effective at targeting and treating cancer cells, as they can be more easily manipulated using external magnetic fields.

Hyperthermia damages the cancer cells by supplying heat from an external source. For this purpose, magnetic nanoparticles can be used to induce a current in the particles using an alternating magnetic field, which generates heat [32]. The shape of the nanoparticles can affect their heating efficiency and the distribution of heat within the tissue. For example, elongated nanoparticles have been shown to produce more efficient heating than spherical nanoparticles [48]. Targeted drug delivery is another potential application of magnetic nanoparticles in cancer therapy. The nanoparticles can be coated with drugs and directed to specific locations within the body using a magnetic field [49]. The shape of the nanoparticles can affect the stability of the drug coating and the ability of the nanoparticles to reach their target location. For example, nanoparticles with a high aspect ratio (i.e., those that are long and thin) have been shown to have improved targeting ability and stability compared to spherical nanoparticles. Cao et al. reported high drug loading and release efficiency of hierarchically nanostructured magnetic hollow spheres for ibuprofen suggesting the role of shape in drug delivery applications [50]. In addition to their use in magnetic drug targeting, MNPs can also be used in other cancer treatment approaches, such as photothermal therapy, in which MNPs are used to convert light energy into heat to destroy cancer cells. The size and shape of MNPs will influence their ability to absorb and convert light energy, as well as their distribution in the body.

Overall, the shape of MNPs plays a critical role in their effectiveness in cancer therapy. By carefully controlling the shape of the MNPs, researchers can optimize their properties and maximize their potential for use in cancer treatment. In particular, the following sections demonstrate the controlled synthesis of MNPs and their functionalization for cancer diagnosis and therapy toward the development of modern medicine. The fabrication of magnetic hybrid nanostructures was accomplished using a variety of synthesis techniques described in detail in the following sections, including polymeric materials, carbon-based materials, noble metals, semiconducting fluorescent nanomaterials, and biomolecules (genetic materials conjugated).

### 2.2. Polymeric–Magnetic Hybrid Nanostructures

**Figure 1:**
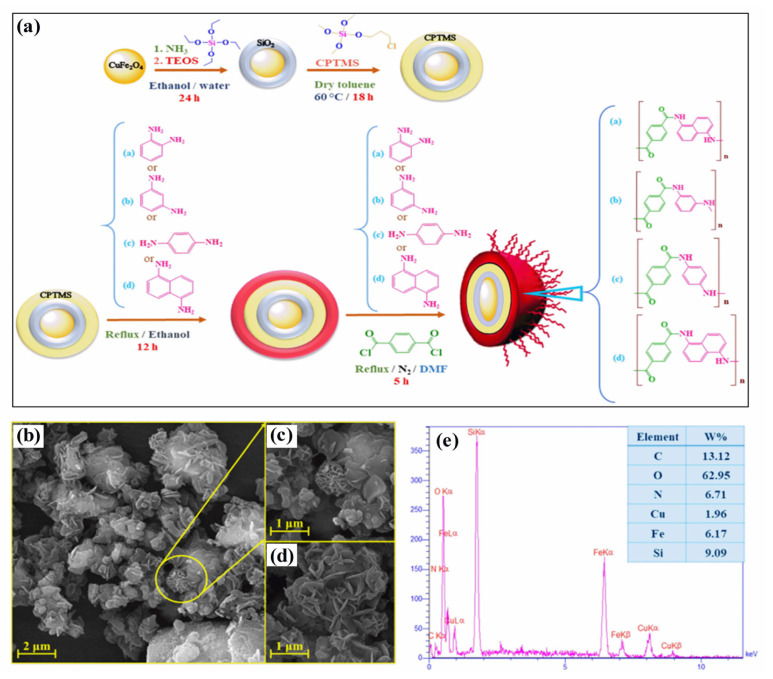
(**a**) Schematic diagram of the formation of CuFe_2_O_4_@SiO_2_-poly(m-phenylene terephthalamide) nanocomposite, (**b**–**d**) SEM images, and (**e**) EDS analysis of CuFe_2_O_4_@SiO_2_-poly(m-phenylene terephthalamide) nanocomposite. Reprinted from Ref. [46]. Copyright 2021 ACS.

Polymer–magnetic hybrid nanostructures have emerged as a promising approach for cancer treatment due to their unique physicochemical properties [51]. These nanostructures are composed of a polymer matrix and magnetic nanoparticles, which can be functionalized with therapeutic agents such as chemotherapy drugs or imaging agents [52]. The magnetic nanoparticles can be attracted to a specific location in the body using an external magnetic field, allowing for targeted delivery of the therapeutic agents to cancerous tumors [53]. Several methods can be used to synthesize polymer–magnetic hybrid nanostructures for cancer treatment. The most common approaches are layer-by-layer assembly, self-assembly, and co-precipitation. Polymer–magnetic hybrid nanostructures are particularly useful for improving the therapeutic efficacy of chemotherapy drugs [54]. In many cases, chemotherapy drugs are insoluble in water, making it difficult to deliver them to cancerous tumors in the desired concentrations. A polymer matrix can improve the solubility of chemotherapy drugs, leading to higher drug concentrations at the tumor site [55]. Furthermore, the polymer matrix can protect chemotherapy drugs from degradation in the body and prevent side effects. Polymer–magnetic hybrid nanostructures can also improve the targeting of therapeutic agents for cancerous tumors [56]. An external magnetic field can be used to attract nanostructures to a specific location in the body by attaching magnetic nanoparticles to their surfaces. Targeted delivery of therapeutic agents can improve the therapeutic efficacy of the treatment by delivering them to the tumor. Recently, CuFe_2_O_4_@SiO_2_-poly(m-phenylene terephthalamide) nanocomposites have been successfully developed by incorporating poly(m-phenylene terephthalamide) onto CuFe_2_O_4_@SiO_2_ nanostructures, as shown in Figure 1a [46]. The SEM images in Figure 1b–d show a unique nanoflower morphology of CuFe_2_O_4_@SiO_2_-poly(m-phenylene terephthalamide) in the present case, which results from the formation of nanoplates oriented in specific directions. EDX spectra also show copper (1.96%), iron (6.17%), and oxygen (62.95%) peaks, which support the presence of CuFe_2_O_4_ cores, as shown in Figure 1e. Spectral analysis confirms the successful polymerization reaction and the formation of *p*-phenylene terephthalamide chains (13.12%), nitrogen (6.71%), and oxygen (0.72%). TEOS and CPTMS shells are responsible for the presence of the silicon peak (9.09%). This hybrid CuFe_2_O_4_@SiO_2_-poly(m-phenylene terephthalamide) nanostructure shows potential for magnetic hyperthermia while exhibiting low toxicity, making this material promising for cancer diagnosis and therapy.

Polymer–magnetic hybrid nanostructures have been developed for cancer treatment, including magnetic liposomes, magnetic nanoparticles, and magnetic polymeric micelles. Several polymeric materials, including poly(glycolic acid) (PGA), poly(lactic acid) (PLA), and their copolymers PLGA, are bioabsorbable, biocompatible, and biodegradable because their ester links can be hydrolyzed by the human body to form metabolites. A magnetic liposome consists of a phospholipid bilayer that encloses a magnetic nanoparticle core, whereas a magnetic nanoparticle consists of a single magnetic layer. Recently, a novel temperature-responsive magnetite/polymer nanoparticle, developed from iron oxide nanoparticles and a block copolymer of polyethylene oxide and polypropylene oxide (PEO−PPO−PEO), has been developed and appears to be an attractive candidate for the treatment of a wide range of biomedical conditions; in particular, drug delivery [57]. Further, the polyhydroxy poly(ethylene glycol) (mPEG) and poly(lactic acid) (PLGA) block copolymers formed micelles loaded with magnetite as nanocarriers for hydrophobic anticancer drugs [58]. In the future, this micelle could be used as a nanocarrier to deliver hydrophobic drugs or to treat cancer. The polymers and structures they are made from retain their characteristics during the reaction and the entrapment of the drugs, in addition to their biocompatibility and biological properties. This research shows that quercetin-loaded magnetic micelles have appropriate properties in terms of loading of dosages, controlled release, and biocompatibility for use in targeted drug delivery systems. Additionally, through a double emulsion method, PLA–PEG–FA magnetic nanoparticles (nanocarrier) loaded with DOX were prepared, which is a potential cancer-combination chemotherapy and hyperthermia nanosystem [59]. In magnetic polymeric micelles, a polymer core is surrounded by a shell of magnetic nanoparticles. Polymeric nanoparticles are small, spherical particles composed of a polymer shell and a magnetic core. They can be used to deliver cancer therapeutics, such as small interfering RNA (siRNA) molecules, which can help to inhibit the expression of specific genes that are involved in cancer development and progression [60,61]. For example, Jaideve et al. reported the synthesis of multi-functional polymeric-magnetic nanoparticles for the treatment of pancreatic cancer and glioblastoma, respectively [62,63]. Gemcitabine and fluorescent iron oxide encapsulated poly(lactide-co-glycolide) conjugated with antibodies for pancreatic cancer treatment and imaging have been shown to effectively inhibit tumor growth in vivo [62]. Iron oxide-based poly-L-lysine-magnetosomes nanoparticles have been shown to exhibit excellent anticancer properties for magnetic hyperthermia treatment of glioblastoma cancer [63]. Polymeric nanofibers are long, thin fibers composed of a polymer and a magnetic material. They can be used in wound dressing and to deliver cancer therapeutics, such as gene therapies, directly to cancerous tissue. Rahmani et al. reported the fabrication and use of curcumin-loaded poly (vinyl alcohol) (PVA)-graphene oxide (GO)-silver (Ag) nanofibers, synthesized through electrospinning, for wound healing in vitro [64]. Chitosan, polyethylene glycol, and polyvinyl alcohol functionalized MgFe_2_O_4_ nanoparticles, synthesized by glycol-thermal method, have shown excellent drug delivery of anticancer drugs (doxorubicin) [65]. Taheri-Ledari reported the synthesis of an iron-based nanotherapeutic. The Fe_3_O_4_ NPs (obtained through the co-deposition technique) were coated with a silica network and encapsulated through PVA taking advantage of H-binding interactions between hydroxyl groups, PVA structure, and Fe_3_O_4_/SiO_2_ surfaces. The therapeutic was reported to be effective in selective drug delivery in ovarian cancer cells [66]. In addition to their use in cancer treatment, polymer–magnetic hybrid nanostructures have also been explored for use in drug delivery and imaging applications. These nanostructures can be functionalized with contrast agents or biomarkers, which can help to visualize cancerous tissue and monitor the progression of the disease through magnetic resonance imaging (MRI) scans. These examples demonstrate the potential of polymer–magnetic hybrid nanostructures for cancer treatment and suggest that these nanostructures may be effective for delivering a wide range of cancer therapeutics to specific areas within the body. Several challenges need to be overcome to fully realize the potential of polymer–magnetic hybrid nanostructures for cancer treatment. One major challenge is the development of effective and stable nanostructures that can withstand the harsh conditions of the body and remain functional over time. Another challenge is the development of effective methods for synthesizing and characterizing these nanostructures, as well as understanding their behavior and interactions within the body. Overall, polymer–magnetic hybrid nanostructures have the potential to revolutionize cancer treatment and diagnosis, and ongoing research in this area is likely to lead to significant advances in the field. However, it is important to note that these nanostructures are still in the early stages of development, and more research is needed to fully understand their behavior and interactions within the body and to optimize their design and function.

### 2.3. Carbon–Magnetic Hybrid Nanostructures

Carbon–magnetic hybrid nanostructures have emerged as a promising approach for cancer treatment [67]. These nanostructures are composed of both carbon-based materials, such as graphene and carbon nanotubes, and magnetic materials, which allow them to be easily manipulated and targeted to specific areas within the body [68]. The potential applications of carbon–magnetic hybrid nanostructures are in the delivery of cancer therapeutics, detection, and diagnosis of cancer [69,70]. The magnetic material allows the nanostructures to be directed to specific areas within the body using an external magnetic field, whereas the carbon-based material can be used to encapsulate and release the therapeutic agent at a controlled rate [70]. The most widely used carbon-hybrid materials are graphene and carbon nanotubes both of which can be used to deliver cancer therapeutics, such as small interfering RNA (siRNA) molecules, which can help to inhibit the expression of specific genes that are involved in cancer development and progression [71]. Song et al. reported the synthesis of core-shell morphology with 10 nm FeCo and poly(ethylene glycol) decorated graphitic carbon coated on FeCo nanoparticles for enhanced cancer imaging and therapy [72]. Graphitic carbon coating on FeCo prevents FeCo leaching and makes the magnetic nanoparticle more stable, whereas poly(ethylene glycol) coating on functionalized MNP surfaces enhances particle stability, dispersibility, and biocompatibility. Moreover, several hollow carbon nanospheres embedded with γ-Fe_2_O_3_ and GdPO_4_ (Fe–Gd/HCS), dual-Fe nanoparticles embedded within synchronized carbon nanostructures, and co-functionalized mesoporous carbon spheres with γ-Fe_2_O_3_ and GdPO_4_ have also been successfully developed and applied for the integration of magnetic resonance imaging and drug delivery [73,74,75,76,77]. Multiwall carbon nanotubes (MWCN) with magneto-fluorescent carbon quantum dots resulted in synergistic effects toward dual-modal targeted imaging [78]. Poly(acrylic acid) functionalized magnetic multiwall carbon tubes and magnetic-activated carbon particles were synthesized and compared as a nanocarrier for drug delivery and cancer lymphatic-node metastasis treatment. The results suggest poly(acrylic acid) functionalized magnetic multiwall carbon tubes are superior for regression and inhibition of metastasis using gemcitabine loading [79]. Dual functioning magnetic MWCN were also prepared by the addition of iron NPs inside the capillary and surface functionalized with gadolinium using the wet chemical method. The developed magnetic carbon structures can be used in MRI imaging and magnetic hyperthermia applications in cancer treatment [80]. Graphene-oxide hybrid with magnetic material could significantly enhance the efficiency of antitumor efficiencies both in vitro and in vivo through magneto thermal effect and reactive oxygen species-related immunologic effect [81]. These studies demonstrate the potential of carbon–magnetic hybrid nanostructures for cancer treatment and suggest that these nanostructures may be effective for delivering a wide range of cancer therapeutics to specific areas within the body.

### 2.4. Noble-Metal-Based Magnetic Hybrid Nanostructures

Cancer treatment using noble-metal-based magnetic hybrid nanostructures is a promising area of research that holds great potential for improving the effectiveness of cancer therapies. Noble metals, such as gold, silver, platinum, and palladium, have unique chemical and physical properties that make them attractive for use in medicine. These properties, combined with the ability to manipulate their size and shape at the nanoscale, make them ideal candidates for use in cancer treatment. The morphology of the as-prepared nanostructures depends on the synthesis conditions used. Based on the synthesis techniques (such as sol-gel, vacuum sputtering, ion implantation, laser ablation, vacuum evaporation, electrochemical method, two-phase method, seed growth method, and other techniques as described earlier), different morphologies such as rod-like, film, spherical, hierarchical, powder, and other morphologies can be attained [82,83,84,85]. The physiochemical properties of noble NPs change as their size and size change [83,84]. A typical example is the change of absorption spectra of gold NPs for spherical (visible region) and rod-shaped (near-infrared region) structures due to the localized surface plasmon resonance effect [86]. Additionally, the unique photothermal and electronic properties of noble metal NPs are a result of the surface-enhanced Raman scattering and metal-enhanced fluorescence effect that can be useful in cancer diagnostic applications [87,88]. The most common structures of noble-metal-based magnetic hybrid nanostructures include nanorods, nanoprisms, nanocages, nanowires, nanocubes, hexagonal sheets, and nanospheres [84,89,90,91,92,93]. Gold nanoparticles are being explored to treat tumors by antitumor drug administration, hyperthermia, and angiogenesis inhibition [94]. When exposed to near-infrared light, these nanoparticles have been shown to have a toxic effect on cancer cells. By incorporating these nanoparticles into nanostructures and targeting them in cancer cells, it is possible to use light to trigger the release of the antitumor drug and kill the cancer cells. This approach, known as photothermal therapy, has shown promising results in preclinical studies and is currently being tested in clinical trials. Additionally, gold-based NPs have been utilized in photothermal chemotherapy to kill cancer cells through cell apoptosis and protein denaturation [95,96]. Song et al. reported the synthesis of hybrid gold nanorods decorated on a mixture of doxorubicin and reduced graphene oxide with excellent photothermal effects. Such a hybrid can effectively be used in hyperthermia and drug delivery applications [97]. Silver nanoparticles have been shown to have a toxic effect on cancer cells and can be used to induce cell death through a process known as apoptosis. In addition, silver nanoparticles have been shown to inhibit the growth of cancer cells, making them potentially useful for preventing the spread of cancer. Bian et al. reported the synthesis of silver nanocages decorated on an octreotide template based on peptide-directed silver mineralization. The particle size and morphology were fine-tuned through the addition of silver nitrate resulting in an optimized surface plasmon resonance behavior. The resulting catalysts were reported to have excellent antitumor properties and photothermal efficiency [98]. Additionally, noble-metal-based magnetic hybrid nanostructures are being used in cancer treatment by magnetic resonance imaging (MRI). Sun et al. reported the synthesis of surface-modified ^64^Cu integrated gold nanorods using polyethylene glycol (PEG) and Cu as surface modifiers for enhanced optical imaging and high targetability [99]. In addition to their use in drug delivery and photothermal therapy, noble nanoparticles are also being explored for use in imaging and diagnosis. By incorporating these nanoparticles into contrast agents, it is possible to enhance the visibility of cancerous tumors during imaging procedures such as magnetic resonance imaging (MRI) or computed tomography (CT). This can help doctors to more accurately diagnose and stage cancer, as well as to monitor the effectiveness of treatment. Palladium-based nanostructures have been reported to enhance the photothermal-related process (used in cancer treatment) efficiency and biocompatibility. The inclusion of functionalized palladium structures through polymers significantly improves water dispersion, physiochemical stability, and biocompatibility. Bharathiraja et al. reported the synthesis of chitosan-modified palladium NPs followed by functionalization with RGD peptide resulting in enhanced efficiency of prepared nanoparticles towards near-infrared region imaging for better tumor diagnosis [100]. Hence, noble-metal-based magnetic hybrid nanostructures show great promise for improving the effectiveness of cancer treatment and increasing the chances of survival for cancer patients. Further research is needed to fully understand the potential of these nanostructures and to optimize their use in the clinic. However, these materials have the potential to significantly impact the way that cancer is diagnosed and treated in the future.

### 2.5. Semiconducting Fluorescent Nanomaterials Magnetic Hybrid Nanostructures

Semiconducting fluorescent nanomaterials are a type of nanomaterial that exhibits fluorescent properties when exposed to light [101]. These nanomaterials can absorb and emit light, making them useful for a variety of applications, including cancer diagnostics [102,103]. The magnetic component of the nanostructure allows it to be guided to the site of the cancer cells using an external magnetic field [104]. Once the nanostructure reaches the cancer cells, the semiconducting fluorescent material can be activated using light, which can then be used to trigger the release of the therapeutic agent [105,106,107]. In photodynamic therapy, the light emitted by the fluorescent nanomaterials activates a photosensitizer, which generates reactive oxygen species (ROS) [108]. These ROS can damage the cancer cells and kill them while minimizing the impact on healthy cells. Semiconducting fluorescent nanomaterial magnetic hybrid nanostructures have several attractive properties for use in cancer treatment, due to their high fluorescence efficiency, tunable emission wavelengths, and ability to be functionalized with a variety of biomolecules [109]. There are several examples of semiconducting fluorescent nanomaterials that have been used in magnetic hybrid nanostructures for cancer treatment. Some of these materials include quantum dots, hybrid nanoparticles, carbon dots, graphene quantum dots, and fluorescent dyes [108].

Quantum dots are nanoscale semiconductor particles that can emit light of different colors depending on their size when excited. They have been used in magnetic hybrid nanostructures for cancer treatment because of their high photostability, which means they can retain their fluorescence over a long period. Hybrid nanoparticles can absorb low-energy light and emit higher-energy light, which makes them useful for photodynamic therapy [110]. They have been incorporated into magnetic hybrid nanostructures for cancer treatment because of their ability to generate ROS when excited. Carbon dots are nanoscale particles made of carbon that have been shown to have fluorescent properties [111]. They have been used in magnetic hybrid nanostructures for cancer treatment because of their biocompatibility and low toxicity. Graphene quantum dots are made of graphene, which is a single layer of carbon atoms arranged in a hexagonal lattice [112]. They have been shown to have fluorescence properties and have been incorporated into magnetic hybrid nanostructures for cancer treatment because of their high stability and low toxicity. Fluorescent dyes are organic molecules that can absorb light at one wavelength and emit it at a different wavelength. Magnetic hybrid nanostructures have been developed for cancer treatment because they can be easily synthesized and have a wide range of emission wavelengths. Fluorescence-based imaging techniques and fluorescence-activated cell sorting (FACS) have been used in a variety of cancer diagnostic applications. Lanthanide-doped nanomaterials are materials that are doped with rare earth elements, such as europium or terbium. These materials can emit light when excited and have been explored for use in cancer diagnosis and imaging. Hence, there are many different types of semiconducting fluorescent nanomaterials that have been used in magnetic hybrid nanostructures for cancer treatment, and more are being developed as research in this area continues.

### 2.6. Biomolecular (Genetic Materials Conjugated) Magnetic Hybrid Nanostructures

**Figure 2:**
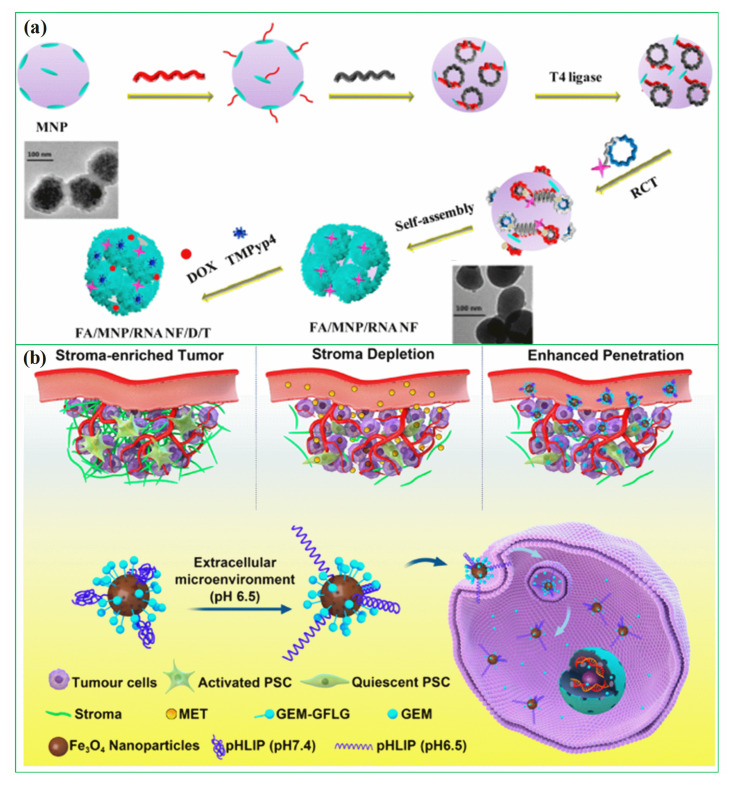
(**a**) Schematic diagram for the development of FA/MNP/RNA NF, reprinted from Ref. [113]. Copyright 2017 ACS; (**b**) A schematic showing gemcitabine-loaded magnetic nanoparticles being developed for pancreatic cancer treatment. Reprinted from Ref. [114]. Copyright 2022 ACS.

In recent years, researchers have been exploring the use of biomolecules conjugated to magnetic hybrid nanostructures for cancer diagnostics [113,114,115]. There are several examples of biomolecules, such as genetic materials, that can be conjugated into magnetic hybrid nanostructures for use in cancer diagnostics [116,117]. A new study was developed a magnetic RNA nanoflower delivery system (RNA NF) has been developed to target cancer therapy, as shown in Figure 2a [113]. Nucleic acid can be conveniently separated by introducing magnetic nanoparticles (MNPs) instead of the traditional nucleic acid structure. MNP/RNA NF modified with folic acid (FA) demonstrated excellent biocompatibility. This FA/MNP/RNA NF is small in size, easy to synthesize, biocompatible, and has high binding affinity and selectivity, making it ideal for drug delivery, imaging of cancer cells, and biomolecule detection. Moreover, gemcitabine-loaded magnetic nanoparticles have been successfully used in the treatment of pancreatic cancer targeted treatments, as shown schematically in Figure 2b [114]. In this work, PEGylated Fe_3_O_4_ nanoparticles with carboxyl groups on the surface were successfully prepared and gemcitabine and peptide (pHLIP) were incorporated to make MET/GEM-MNP-pHLIP. A new cascade treatment for pancreatic cancer utilized MET in an innovative way that could have greatly improved therapeutic outcomes.

Biomolecules, such as DNA and proteins, can be conjugated into magnetic hybrid nanostructures to create contrast agents for use in cancer diagnostics. These nanostructures, which are typically composed of a magnetic core surrounded by a shell of biomolecules, can be used to enhance the visibility of cancerous tumors during imaging procedures such as magnetic resonance imaging (MRI) or computed tomography (CT). By incorporating DNA into the nanostructures, it is possible to enhance the sensitivity and specificity of the diagnosis, as the DNA can bind specifically to cancer-associated genes or proteins. RNA, the chemical cousin of DNA, can also be conjugated to magnetic hybrid nanostructures and used to detect specific genetic mutations associated with cancer. Proteins, such as enzymes and antibodies, peptides, and short-chain amino acids, can be conjugated to magnetic hybrid nanostructures and used to detect specific proteins or biomolecules associated with cancer. This can help to diagnose cancer in its early stages, as well as to monitor the effectiveness of treatment. One of the main advantages of using biomolecules conjugated to magnetic hybrid nanostructures for cancer diagnostics is their ability to specifically target cancer cells. By designing the biomolecules to bind to specific receptors or biomarkers found on the surface of cancer cells, it is possible to create contrast agents that are preferentially taken up by tumor cells. This can help to improve the accuracy of cancer diagnosis, as well as to monitor the effectiveness of treatment. Another potential use of magnetic hybrid nanostructures conjugated with biomolecules in cancer diagnostics is using biosensors. These nanostructures can be designed to detect specific biomolecules that are associated with cancer, such as specific proteins or genetic mutations. By detecting these biomolecules, it is possible to diagnose cancer in its early stages, when it is most treatable.

Protein-magnetic hybrid nanostructures are also being explored for use in cancer diagnosis. These nanostructures can be designed to specifically target cancer cells and can be used to detect the presence of cancerous tumors with MRI or other imaging techniques. By incorporating proteins into the nanostructures, it is possible to enhance the sensitivity and specificity of the diagnosis, as the proteins can bind specifically to cancer-associated markers or proteins. Overall, the use of biomolecules conjugated to magnetic hybrid nanostructures holds great promise for improving the accuracy and efficiency of a cancer diagnosis. Further research is needed to fully understand the potential of these nanostructures and to optimize their use in the clinic. However, these materials have the potential to significantly impact the way that cancer is diagnosed and treated in the future.

## 3. Cancer Diagnosis

Early diagnosis of cancer diseases is critical to receiving accurate treatment promptly [118]. Magnetic nanomaterials are being used to diagnose a variety of cancer diseases [119]. The development of more sensitive and accurate diagnostic tools allowed efficient and early diagnosis to be achieved. Several imaging techniques are being developed for cancer diagnosis, including magnetic resonance imaging (MRI), magnetic nanoprobes, magnetic nanoparticles for multimodal image acquisition, magnetic-optical imaging probes, and magnetic biosensors. A cancer diagnosis system has been developed by combining a variety of magnetic nanoparticles with hybrid nanostructures. A magnetic hybrid nanostructure (MHNs) can be constructed in five main types: diamagnetic (Au, Ag, and Cu), paramagnetic (Mg, Li, tantalum, and Gd), ferromagnetic (Co, Ni, and Fe), antiferromagnetic (CoO, MnO, and NiO), and ferrimagnetic (Fe_3_O_4_ and γ-Fe_2_O_3_) [120]. The size, shape, crystalline structure, and chemical composition of MHNs have a significant impact on their chemical and physical properties. The following section provides in-depth information on MHNs involved in various diagnosis systems [44]. In particular, the diagnosis system is integrated with AI technology to enhance its performance. The purpose of this section is to discuss recently published articles on the integration of artificial intelligence with cancer diagnosis systems.

### 3.1. Magnetic Resonance Imaging (MRI)

Magnetic resonance imaging (MRI) provides detailed images of anatomical structures in three dimensions. Detection, diagnosis, and monitoring of disease are often carried out using it. Living tissues are made of water that contains protons that can be excited and detected by sophisticated technology. MRI images can be classified as longitudinal (T1)- or transverse (T2)-weighted images based on relaxation pathways [121]. Clarification and interpretation of MRI images can be improved with contrast agents. MNPs are commonly used as contrast agents for T2, whereas paramagnetic complexes are used for T1 [122]. In general mechanism, a radio frequency pulse is applied to the human body in a static magnetic field in order to cause magnetic resonance (MR) by excitation of hydrogen protons in the body. Protons generate an MR signal when they relax after the pulse is stopped. A variety of procedures must be performed in order to generate MR signals, including receiving, spatial coding, and reconstruction of images. MR signals are primarily generated by spin characteristics. In most cases, 1H is one of the most ideal elements for nuclear MRI and NMR spectroscopy because of its inherent sensitivity [123]. The injection of MRI contrast agents caused the resonance time of the tissue to be shortened, the contrast signal difference is increased, and imaging contrast and clarity are improved. It is therefore possible to alter the water proton relaxation rate in tissues and shorten the relaxation time of protons within water molecules which leads to determining the physiological differences between normal and abnormal states. Recently, several studies have been conducted on MHNs in (T2)-weighted MRI fields. MHNs made of various carbon–magnetic, polymer–magnetic, fluorescent–magnetic, and various metal alloys, such as FeCo and FePt, serve as MRI contrast agents [124].

**Figure 3:**
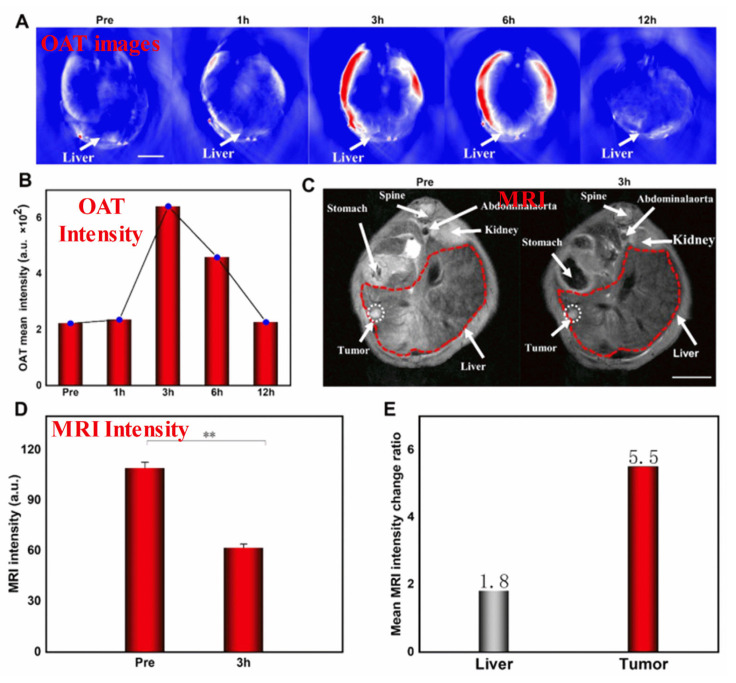
(**A**–**E**) In-vivo OAT−MRI studies. In this study, Fe_3_O_4_@Au-PEG-EpCAM as a contrast agent, and HepG2 as the tumor cell. Reprinted from Ref. [128]. Copyright 2022 ACS. ** *p* < 0.01.

Specifically, SPIONs conjugated with monoclonal antibody C95 (SPIONs-C595) and c(RGDyK)-PDA-SPIONs have recently been successfully used as T2-weighted MRI contrast agents for detecting breast cancer (MCF-7) and liver cancer cells, respectively [125]. Further, the use of poly(ethylene glycol) as a stabilizing agent enables the development of SPIONs with a smaller diameter, which have extraordinary potential as real-time contrast agents for MRI and as continuous tumor monitoring agents. SPIONs are useful in theranostic applications and advanced MRI examinations. To develop novel MHNs, carbon, metal, and polymer nanocomposites can be incorporated into SPIONs to enhance their MRI performance. In recent studies, macrophage-mediated delivery of Fe@Fe_3_O_4_-DHCA MHNs has been examined to assess the impact on MRI [126]. Figure 3 shows MRI images obtained from a 1 T scanner of 4T1 tumor-bearing mice before (control) and after intravenous injections of RAW264.7 cells loaded with Fe@Fe_3_O_4_-DHCA. Notably, the Fe@Fe_3_O_4_-DHCA loaded RAW264.7 cells exhibit obvious T2-weighted MRI imaging performance and can deliver Fe@Fe_3_O_4_-DHCA nanoparticles to the tumor with a high degree of enrichment. In addition, Ge et al. prepared Fe_3_O_4_@Au composite MHNs for contrast agents in MRI. They were shown to be efficiently absorbed, capable of preferentially targeting U251 cells, and effective in targeting gliomas in vivo [127]. This demonstrates that they can be used to diagnose gliomas in vivo. Furthermore, Fe_3_O_4_@Au nanoparticles were further developed as an HCC-targeted nanoprobe for optoacoustic tomography (OAT), MRI, and photothermal sensing [128]. Specifically, Fe_3_O_4_@Au is used in the dual model system of OAT-MRI to detect HepG2 tumors at different times. The target and metabolic ability of the contrast agent were verified by injecting Fe_3_O_4_@Au-PEG-EpCAM into the caudal vein. It was found that the average OAT signal at the tumor site peaked after 3 h and then decreased after 12 h as nano drugs accumulated, as shown in Figure 3A,B. There was a significant correlation between orthotopic tumor signal intensity and time, suggesting that OAT imaging and tumor PTT are best performed 3 h after injection. A T2-weighted MRI was conducted on HCC mice to investigate the feasibility of using NPs as enhanced MRI agents, as shown in Figure 3C. After the injection of targeted Fe_3_O_4_@Au-PEG-EpCAM, the T2 signal was enhanced. The results of this study suggest that Fe_3_O_4_@Au-PEG-EpCAM may be highly effective for detecting micro-HCC by OAT−MRI. This probe can be used to improve cancer diagnosis. However, such large particles cannot be used in clinical applications. To address this issue, a number of recent studies have explored developing smaller-sized MHNs that can be used as thermal treatment contrast agents guided by MRI. The study found that nanoparticles with a smaller diameter and a low copper content such as Cu_0.08_Zn_0.54_Fe_2.38_O_4_ possessed long-term colloidal stability in water due to the effective coating of non-degraded poly(ethylene glycol) [129]. Further, PEGylated Cu_0.08_Zn_0.54_Fe_2.38_O_4_ with a smaller diameter of below 5 nm was used as the T2 weighted MRI contrast. In spin-echo T2-weighted MR images, temperature and image intensity were strongly correlated for aqueous phantoms embedded with Cu_0.08_Zn_0.54_Fe_2.38_O_4_ MHNs. The study concluded that the MRI thermometry can be improved by using Cu_0.08_Zn_0.54_Fe_2.38_O_4_ MHNs as contrast agents. The use of these MHNs as MRI contrast agents is beneficial for advanced examinations, such as imaging and theranostic applications.

T2-weighted images provide high endogenous contrasts, whereas clinical imaging requires exogenous contrast agents with positive T1 contrast enhancement to increase image intensity. Gadolinium-based contrast agents (GBCAs) are the only FDA-approved MRI contrast agents. Potential side effects of GBCAs include kidney failure, hypertension, and nephrogenic system fibrosis. SPIOs have long been used for T2 MRI contrast agents with a core size between 20 and 50 nm. Magnetic nanoparticles can be active in T1 MRI contrast agents when their size is reduced to less than 5 nm. A smart contrast agent for T1 MRI can be developed with ultra-small superparamagnetic iron oxide nanoparticles (uSPIOs). Enhanced T1 MR contrasts are possible with a small hydrodynamic diameter, which ensures optimal pharmacokinetics and delivery profiles to tumors. The T1 relaxation process was enhanced in ultra-small magnetic nanoparticles by two factors: (i) a smaller diameter of the magnetic nanoparticle enhanced the exposure of multiple Fe^2+^/Fe^3+^ ions to water protons that diffused through the hydrophilic layer and shortened their longitudinal relaxation time, and (ii) short correlation times of ultra-small magnetic NPs also favored T1.

**Figure 4:**
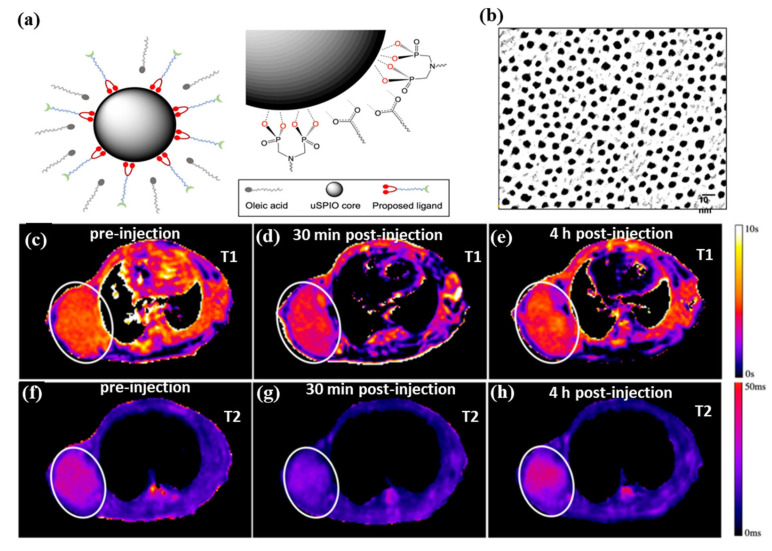
(**a**) Diagram illustrates the process of exchanging ligands and uSPIOs, (**b**) TEM image of uSPIOs, (**c**–**e**) T1 mapping, and (**f**–**h**) T2 mapping of the uSPIOs in the mouse at a different time interval. Reprinted from Ref. [130]. Copyright 2021 ACS.

Figure 4a,b shows water-soluble and ultra-small superparamagnetic iron oxide nanoparticles (uSPIOs) that have been synthesized via functionalization of a hydrophilic layer to allow further modification for targeted delivery and therapeutics [130]. As an MRI contrast agent, the smaller diameter hydrophilic uSPIOs were successfully used. A tumor-bearing mouse model BT-474 (N = 4) was injected with uSPIOs to evaluate uSPIOs distribution and clearance in the tissues. As shown in Figure 4c–h, in both T1 and T2 contrasts, uSPIOs induced approximately 15% enhancement around 30 min to 1 h, which recovered to about 99% four hours later. Approximately 1 h after injection, the greatest T1 contrast enhancement was observed as compared to T2. In particular, uSPIOs with smaller diameters possessed two to three empty orbits per iron (Fe^2+^ or Fe^3+^), which induce efficient T1 relaxation. A number of researchers have also developed iron-based contrast agents for T1 MRIs. The PEGylated Fe^3+^-MelNPs worked in mice with healthy spleens and livers and showed bright signals upon intravenous injection [131]. On the other hand, contrast agents have difficulty reaching the brain and intracranial tumors due to the blood–brain barrier (BBB). The T1-weighted MRI can visualize intracranial brain tumors after intravenous injection of oligosaccharide-coated sub-5 nm ultrafine magnetic iron oxide nanoparticles (uIONP) [132]. The T1-enhanced MRI contrast gradually increased after injection of uIONP, showing a time-dependent brain tumor uptake. The validations showed that uIONP remained compartmentalized in tumor blood vessels at the earliest time point (20 min), followed by extravasation. This was mainly due to the size advantage of sub-5 nm in the EPR-driven process that led to the delivery and accumulation of uIONP in the intracranial tumor. The use of uIONP-based MRI is a promising approach for molecular imaging of brain tumors, which is essential for guiding treatment choices. In addition, SPIONs (diameters between 11 nm and 22 nm) showed a strong T1 contrast enhancement (brighter contrast) in 0.13 mT ultra-low field MRI [133]. Compared to conventional ULF MRI, SPION-based T1-weighted MRI has the advantages of a higher signal, shorter imaging time, and biocompatible non-toxic agents based on iron oxide. The approach could become a functional imaging approach, like PET, despite its low spatial resolution. Further, supramolecular amorphous iron oxide (SAIO) is designed as a new type of contrast agent for high-resolution MRI with ideal T1 contrast effects [134]. It consisted of a supramolecular polysaccharide core patched with iron oxide. Ferric oxide hydrous with amorphous Fe^3+^ properties is essential for optimal T1 MRI contrast with a similar relaxation coefficient ratio (r_2_/r_1_) as gadolinium (Gd). Due to advances in MRI hardware and pulse sequences, SAIO could be an ideal contrast agent for quantitatively evaluating the morphology of various blood vessels, such as cerebral, peripheral, and coronary vessels. There is strong evidence that MHNs that are larger than 10 nm are effective as a T2 MRI contrast agent, and the use of MHNs that are smaller than 5 nm is effective as a T1 MRI contrast agent. MHNs have proven to be effective as both (T1 and T2 MRI contrast) in terms of both their size and surface functionalization as long as both are optimally tuned. Moreover, artificial intelligence (AI) is well suited to MRI due to its inherent soft-tissue contrast, variety of structural and physiological acquisition protocols, and diagnostic capabilities [135]. Notably, MRI will transform into a new era of quantitative imaging with AI by utilizing these large data structures to revolutionize its largely qualitative clinical applications [136]. Recently, MRI techniques were successfully used to diagnose lung cancer, liver cancer, prostate, and breast cancer cells, and AI was also integrated into these techniques, allowing them to be integrated into multidisciplinary applications allowing patient-specific medicine to be personalized [137,138,139]. This investigation clearly demonstrated that AI should be integrated into designing the magnetic materials for MRI imaging and the obtained MRI images successfully enhance the diagnostic capabilities [140].

### 3.2. Magnetic Fluorescent Imaging Probes

**Figure 5:**
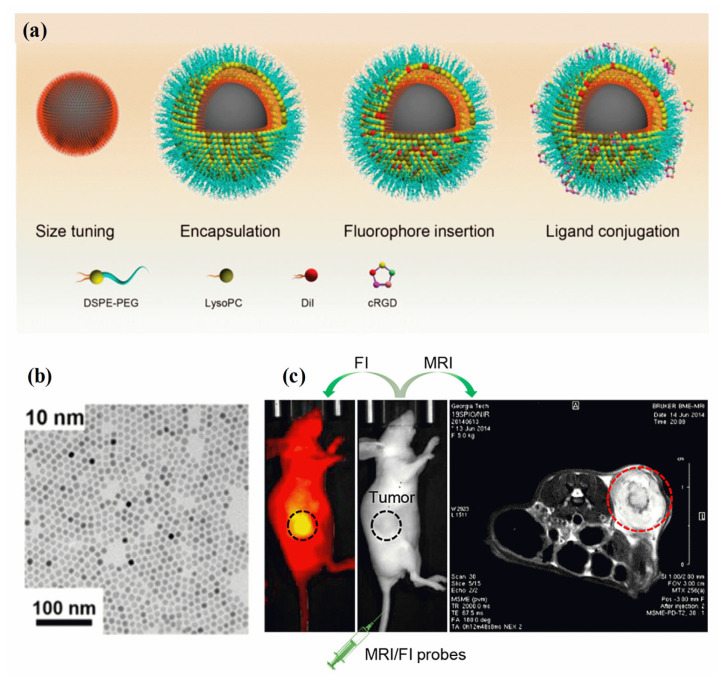
(**a**) Schematic diagram of MRI/FI nanoprobes using Fe_3_O_4_ NPs, (**b**) TEM image of Fe_3_O_4_ NPs, and (**c**) the use of MRI/FI nanoprobes for imaging tumor-bearing mice. Reprinted from Ref. [145]. Copyright 2020 ACS.

A fluorescence image is non-invasive, highly sensitive, non-radiotoxic, low-radiation, and non-radiotoxic. Fluorescent imaging has the advantages of high sensitivity, real-time imaging, and open timing. Meanwhile, MRI imaging offers several benefits, including a high resolution and depth of field that can be adjusted as needed [141]. Analyzing the molecular composition and anatomy of a body can be achieved using MRI techniques. A synergistic imaging tool combining MRI and fluorescent imaging for precisely visualizing and demarcating structural/functional details before cancer treatment. Through the combination of magnetic and fluorescent (FI) properties in MHNs, high-performance cancer diagnosis and treatment can be achieved [142]. There are several clinical imaging applications for these two imaging modalities, including tissue biopsy, disease detection, cancer diagnosis, and pre- and intraoperative imaging. The surface chemistry and geometry of a single entity require the use of MHNs-based MRI contrast agents, fluorophores, coating polymers, and target ligands [143,144]. It is possible to assess cancer disease at different spatial scales and resolutions using MRI and FI probes (MRI/FI) in combination. As demonstrated in Figure 5a–c, MRI/FI probes have been developed with Fe_3_O_4_ nanoparticles and fluorescence agents [145]. Fe_3_O_4_ nanoparticles encapsulated in phospholipids, physically adsorbing dialkylcarbocyanine dyes, and surface bioconjugation of targeting ligands were demonstrated to deliver high contrast in both ex vivo and in vivo MRI and high-resolution fluorescence imaging of cancer. Three distinct molecular assembly processes are enabled by the lipid layer in the MRI/FI nanoprobes: encapsulation of magnetite nanocrystals, control of size, and adsorption of dialkylpyrrolidones. In addition, the EPR effect allows sufficient penetration of tumor vessels via lipid-assembled MRI/FI probes with sizes ranging from 20 nm to 50 nm (with coating). A wide range of biomedical applications can be optimized using this method for MRI/FI probes. A sub-5 nm nanoprobe conjugated to phenothiazine derivatives (PZD) has been designed and prepared for effective T1−T2 magnetic resonance multimodal imaging of A plaques [146]. It is well known that UFNPs@PEG/PZD has excellent properties of r1 and r2 relaxivities in addition to being highly binding to plaques. Hence, these results offer promising ultrasmall nanoplatforms for the development of early detection of Alzheimer’s disease using multimodal imaging techniques. A thiol-functionalized CuFeSe_2_ nanocrystal exhibits broad NIR absorbance in the range of 500 to 1100 nm and magnetic properties that could successfully be used to fabricate MRI/FI probes for computed tomography imaging-guided photothermal therapy of cancer [147]. Researchers found that Fe_3_O_4_ nanoparticles and a redox-responsive polymer ligand (RMNs-HSA-Cy5.5) can be used as MRI/FI probes for the detection of breast cancer [148]. The test also confirmed that tumors accumulate more transport protein in real-time. Further, HP-β-CD functioned Fe_3_O_4_/Carbon NPs were used as high-performance dual-modal MRI/FI probes to characterize tumor accumulation, size, and boundary, and to monitor their biodistribution [149].

In order to introduce a more cost-effective and less-toxic multimodal contrast agent for MRI/FI probes replacing conventional heavy metal containing Gd-DOTA, carbon-decorated ferrite nanodots (CDs@MNFs) MHNs were developed [150]. Surface-engineered ferrite nanodots generate T1 and T2 MRIs along with fluorescence emission without applying labels. It has been shown that CDs@MNFs are potentially cost-effective multimodal imaging agents with negligible toxicity and significant contrast enhancement with stimuli-responsive drug release kinetics. Particularly, CDs possess exceptional characteristics such as photostability, superior physical and chemical stability, tunable photoluminescent behavior, and enhanced water solubility. Hence, the development of various carbon dots (CDs) and graphene quantum dots (GQDs) from various non-toxic biological sources in order to develop new MRI/FI probes is of great importance to the future of medical imaging technology.

Moreover, magnetic NPs with semiconducting quantum dots (SQDs)-based MHNs have been widely used for multimodal MRI/FI nanoprobes for cancer diagnosis and treatment. It is possible to modify the surface of SQDs easily, making them remarkably photostable for fluorescent labeling, as compared to polymers and biological agents. When SQDs are combined with magnetic properties, they can be used for magnetic resonance imaging as well as fluorescence imaging toward the diagnosis of cancer. A water-dispersible and magnetic CdTe/ZnS mQD can be selectively incorporated with ferrous ions in either the core or shell [151]. In particular, shell doping allows for the customized design of paramagnetic SQDs with biocompatible and modifiable surfaces. Cytotoxicity assays with HepG2 cells show that N-acetyl-L-cysteine is a sufficient organic ligand to prevent toxic metal ion leakage of CdTe/ZnS mQD [152]. In vitro fluorescence and magnetic resonance (MR) imaging of cancer cells can be performed by using aromatic and amphiphilic copolymer nanoprobes encapsulated with CdSe@CdS and Fe_3_O_4_-based MRI/FI nanoprobes. Additionally, photothermal therapy and MRI/FI nanoprobe-labeling detection of cancer cells was achieved with nanoplatforms integrated with Fe_3_O_4_ clusters@CdTeS quantum dots (QDs) embedded in mesoporous SiO_2_ [153]. CdTeS QDs were used as a fluorescence-labeling agent in conjunction with the Fe_3_O_4_ cluster core. Particularly, CdS, CdTe, ZnS, and CdTeS-based quantum dots have been successfully combined with magnetic nanoparticles for the development of multimodal MRI/FI nanoprobes. For the fabrication of successful MRI/FI nanoprobes using semiconducting magnetic nanohybrids, the following points must be taken into account: (i) appropriate synthesis strategies need to be developed for the fabrication of magnetic cores and semiconducting quantum dots shells, (ii) magnetic and semiconducting quantum dots must have superior physical and chemical stability, and (iii) magnetic and semiconducting quantum dots are required to be biocompatible with tissues and to be easily regenerated after diagnosis and treatment.

### 3.3. Magnetic Biochips

**Figure 6:**
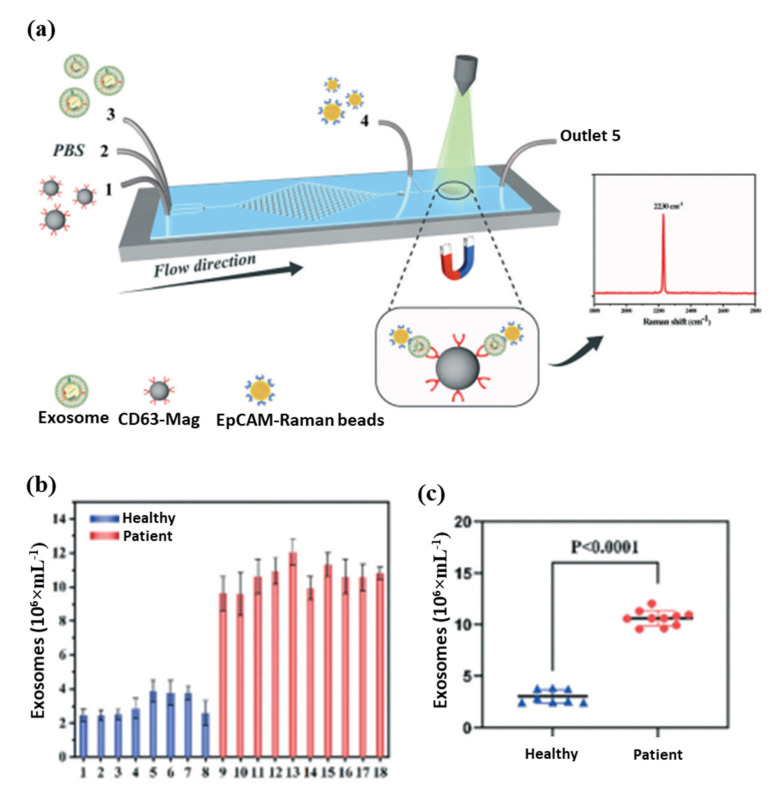
(**a**) Schematic diagram of exosome detection and capture using a microfluidic Raman chip, (**b**) detection of exosomes in clinical serum directly using a microfluidic Raman chip, and (**c**) analysis of clinical samples using dot plots [154].

In biomedical research and clinical cancer diagnosis, microfluidic biochips are excellent tools for analyzing liquids. The magnetic separation device consists of a miniature microfluidic chip with a dense array of magnetic pores [154,155]. It offers a high throughput and efficient release of captured tumor cells that have been labeled with magnetic nanoparticles and have been captured from whole blood. A magnetic sifter and biochip approach was successfully used to isolate and analyze circulating tumor cells from patients with lung cancer. Further, an anti-CD63 magnetic nanoparticle-based microfluidic Raman biochip for exosome isolation and analysis has been developed, as shown in Figure 6a [154]. With EpCAM-functionalized Raman beads, exosome samples can be analyzed within one hour with a quantitative signal at 2230 cm^−1^. From these analyses, exosomes isolated from the serum of PCa patients were higher than those from healthy patients, as shown in Figure 6b. The microfluidic Raman chip discriminated well between PCa patients and healthy controls as shown in Figure 6c. This microfluidic Raman chip provides a promising method for diagnosing PCa. In a recent study, an on-chip magnetic separation system was developed to help researchers efficiently extract sEVs from cell culture supernatants, which is essential for later biological research and cancer diagnosis in the future [156]. A label-free magnetic separation of nanobacterial samples is only possible if (i) a high magnetic force is applied to achieve nanoscale resolution and (ii) the ferrofluid is made biocompatible. These are the key factors to achieving label-free magnetic separation of nanobiological samples.

Recently, a new type of microfluidic chip based on magnetic nano chains is being developed to separate biomaterials and diagnose cancer. Especially, polydopamine complex MHNs and Fe_3_O_4_ NPs were used to fabricate nano chains-based microchips (MiChip) [157]. Thus, target-specific capture antibodies (Ab-I) and thiolated poly(ethylene glycol) (PEG) can be sequentially used to functionalize nanochains (Magchains). In its first proof-of-concept application, MiChip can simultaneously detect three serum protein biomarkers: carcinoembryonic antigen (CEA), AFP, and prostate-specific antigen (PSA), all of which are commonly used in clinical tests for colorectal, hepatocellular, and prostate cancers, respectively. The Magchains can capture 91% CEA, 90% AFP, and 95% PSA based on off-chip enzyme-linked immunosorbent assays. High recovery and negligible crosstalk are key to the realization of multiplexed assays in Magchain. Additional benefits include the ability to accommodate multiple channels on one chip, which allows for increased translational throughput and spatial resolution for cancer detection at an early stage. Magnetic nanoparticles may therefore be used to manufacture microchips that are capable of detecting cancer more efficiently using an early detection system. The development of a low-cost magnetic microchip system may make it possible to detect cancer at an earlier stage. In addition, this design is being discussed for several potential improvements in the future. In today’s life sciences, artificial intelligence (AI) provides a myriad of promising opportunities. Analyzing massive datasets generated by biotechnology systems can be greatly benefited by using AI methods. Analysis of microfluidic data, such as that generated by reaction chambers, arrays, and positioning systems, is not always successful. AI methods are significantly more efficient at analyzing huge datasets obtained from high-throughput and multiplexed microfluidics compared to microfluidics, both of which improve experimental methods and reduce cost and scale. Recently, microfluidics-based imaging flow cytometry with AI-integrated technology had a significant role in the investigations of cancer cell imaging [158]. Especially, HL60, MOLT, and K562 were successfully classified with a CNN trained on ImageNet as the non-medical image database [159,160]. A deep learning technique was used to classify the cell lines above, which outperformed traditional systems. A cost-effective method for screening cancer in low-resource settings was possible with this method. In addition, SW-480 epithelial cancer cells and OT-II WBCs could be identified with greater than 95% accuracy using deep CNNs to process flow cytometry waveforms [161]. The neural network was proposed to classify cells within milliseconds and provide instantaneous results. It offers a rapid, label-free way of sorting cells. The next big advance in this field is the combination of microfluidics and artificial intelligence (AI). Microfluidic regeneration will be greatly impacted by AI since it opens up a wide variety of new possibilities in various aspects of microfluidics.

### 3.4. Magnetic Biosensors

**Figure 7:**
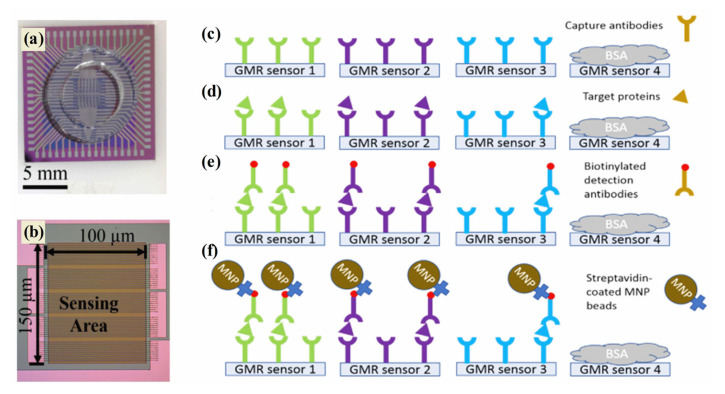
(**a**) GMR bio sensing chip, (**b**) 10 GMR sensor strips connected in parallel, and (**c**–**f**) assay sequence: capture antibodies (**c**), target proteins bound to capture antibodies (**d**), biotinylated detection antibodies (**e**), and GMR signals monitor (**f**). Particularly on the GMR, green, purple, and blue are associated with CA125 II, HE4, and IL6 [166].

In order to provide a reliable and accurate method of cancer detection as well as to deliver viable diagnostics and prognoses, an effective tool is therefore required. Magnetic sensing techniques demonstrate a variety of advantages, making them a promising technology for cancer diagnostics [162]. In addition to their selective segregation and target-capturing properties, magnetic nanoparticles are currently being studied for their use in efficient segregation [163]. The development of innovative magnetic sensing methods for detecting a wide range of biomolecular targets has been the subject of significant cancer diagnosis research over the past two decades [164]. Typically, magnetic nanostructures are used to develop giant magnetoresistance (GMR) biosensors, biomarker detection sensors, and electrochemical sensors for cancer detection. In 1988, Fert and Grünberg discovered the GMR effect by alternating ferromagnetic and non-magnetic layers in multilayer structures [165]. A GMR nanosensor offers the advantages of both technologies—sensitivity and versatility in addition to a low price and quick test time. A change in resistance occurs in the magnetic stack structure of the GMR nanosensor which detects biomolecules such as proteins and DNA. The labeling system of GMR biosensors is robust due to the use of magnetic nanoparticles (MNPs). Magnetic biosensors such as GMR are more sensitive to low levels of background noise since biological tissues and fluids are non-magnetic or diamagnetic. The intrinsic advantages of GMR biosensors are being used to develop a growing array of cancer diagnosis applications. A recent study in Figure 7a–f demonstrated that three biomarkers (CA125 II, HE4, and IL6) were successfully detected by GMR in late-stage serous ovarian cancer patients [166]. A portable prototype of the system provides high-sensitivity multiplex assays capable of serving as platforms for many diseases, including ovarian cancer. This portable system detected CA125 II, HE4, and IL6 multiple times, with limits of detection (LOD) below 3.7 U/mL, 7.4 pg/mL, and 7.4 pg/mL, respectively. Furthermore, commercial magnetic NPs were hybridized with gold nanoparticles to develop MR-based sensors that detect human IgG in water with high sensitivity. The deduction limit was estimated to be 13 pM (2 ng mL^−1^) [167]. MR biochips are also suitable for multiplexed analysis, since they are portable, making them ideal for point-of-care devices for cancer therapy. Further, maghemite NPs were combined with giant magneto-impedance sensors (GMIs) to diagnose rat prostate cancer cells (Mat Ly Lu) [168]. An optical microscope confirmed maghemite NP accumulation in the cells, whereas an X-ray fluorescence measurement quantified the NPs per cell. The recent investigation focused that the spindle-like Fe_3_O_4_, Fe_3_O_4_@Ag MNPs, and ferrites nanostructures that were successfully applied in the GMR devices for the diagnosis of cancer [169,170,171].

Moreover, GMR biosensors are intrinsically more sensitive than optical biosensors given that biological specimens are non-magnetic (or diamagnetic). It is therefore expected that a biological matrix would generate negligible magnetic background noise. However, GMR sensors require specialized magnetic wafers, which makes them more difficult to implement than other magnetic detectors, such as magnetic immune sensors and electrochemical sensors. On the other hand, combining GMR biosensor arrays with CMOS electronics facilitates high-resolution brain imaging and multiplexed bio-assays. A growing list of real-life biomedical applications is being explored by researchers using GMR biosensors due to their unique characteristics. Additionally, magnetic nanomaterial-based electrochemical sensors are widely available and play an influential role in cancer diagnosis. An electrochemical biosensor is a fast, cost-effective, and miniaturized point-of-care testing method (POCT) for cancer diagnosis [172]. Recently, a biosensor that detects prostate cancer via PCA3 biomarkers has been developed via electrochemical and impedance methods. Many protein and gene-based biomarkers have been used in clinical studies, such as cancer antigens (CA19–9, CA125, and CA15–3), AFP, HER2, HER4, APT, MUC 1, and ILs. Each of these biomarkers has been successfully detected by electrochemical sensors. To develop electrochemical biosensors for cancer diagnosis, amperometry, potentiometry, voltammetry, and electrochemical impedance spectroscopy (EIS) techniques are widely used. Especially, the various magnetic nanostructures play a key role in the development of electrochemical biosensors that can detect cancer biomarkers, antigens, antibodies, and proteins [173,174]. The recent development of magnetic heterogeneous hollow nanorods containing α-Fe_2_O_3_/Fe_3_O_4_-Au was successful in detecting tumor antigen 125 using voltammetry techniques [175]. In addition to its low cost and convenience of preparation, the reported electrochemical aptasensor is convenient to use, indicating that it has potential clinical applications. A suitable aptamer can be selected to extend the electrochemical aptasensor to other tumor markers. An aptacytosensor based on CoFe_2_O_4_@Ag magnetic nanohybrids and MXenes has shown excellent potential for monitoring the progression of cancer at an affordable cost through blood cell monitoring [176]. MXene nanosheets functionalized with CoFe_2_O_4_@Ag-HB5 were used to capture SK-BR-3 cells and monitor them electrochemically. HER2-positive cancer cells in the blood can be detected within 75 min with this label-free, sensitive, selective, and simple MXene-based cytosensor. A Fe_3_O_4_@SiO_2_@Au MNC-based electrochemical immunosensor has also been successfully used to deduce serum human epididymis protein 4 [177]. Recent studies have demonstrated that functional magnetic nanoparticles (Fe_3_O_4_ NPs) are capable of increasing miRNA detection sensitivity [178]. Especially, the Fe_3_O_4_ NPs carry many redox signals, enabling dual signal amplification toward hairpin capture probes. In addition, this sensor model allows for the simultaneous detection of different types of miRNAs by using distinct electrical signal molecules. Recent works demonstrated that nanoparticulated materials with the formula MFe_2_O_4_ (M = Mg, Ni, Co, Mn, Cu, and Zn) are capable of demonstrating differentiated performance in the development of electrochemical biosensors for cancer diagnosis. Importantly, nanoparticles based on nickel ferrite (NiFe_2_O_4_) have been a key component of highly sensitive and selective electrochemical sensors. A NiFe_2_O_4_ spinel contains (Fe^3+^)_Td_(Ni^2+^Fe^3+^)_Oh_O_4_, in which Td and Oh represent the tetrahedral and octahedral sites, respectively. A dopant can exchange host ions with an appropriate dopant (Ni) in order to boost sensor performance. This unique structure renders NiFe_2_O_4_ an excellent electrode material for the sensitive detection of p53 and other ovarian cancer markers in serum samples [179,180,181]. Further, nanocomposite Ag-CoFe_2_O_4_-GO has been employed as an interface for unlabeled electrochemical immunosensors to detect tumor markers, such as a carcinoembryonic antigen. A dispersion of CoFe_2_O_4_ on the surface of GO prevents agglomeration and increases conductivity. With the excellent electrochemical activity of Ag NPs, not only can redox reactions be produced, but also electrochemical signals can be enhanced [182]. In addition to having high selectivity and good sensitivity, the constructed immunosensor is capable of detecting CEA rapidly. The electrocatalytic activity of CFCPE at electrode surfaces oxidized OXY and COD with remarkable efficiency. Differential pulse voltammetry was used to study the electrochemical oxidation of OXY and COD at the CFCPE. Clinical practice and medical research can use it to detect carcinoembryonic antigens. Oxycodone (OXY) and codeine (COD) can also be detected using carbon paste electrodes modified with other ferrite-based materials of CoFe_2_O_4_ nanoparticles (CFCPE) [183]. The electrocatalytic activity of CFCPE at electrode surfaces oxidized OXY and COD with remarkable efficiency. Differential pulse voltammetry was used to study the electrochemical oxidation of OXY and COD at the CFCPE. In fact, since the CoFe_2_O_4_ can be prepared easily and the excipients do not interfere with the determination of analytes, this presented method represents an excellent alternative to quality control tools and shows excellent analytical performance in determining OXY and COD simultaneously. Meanwhile, several ferrites, including CuFe_2_O_4_, ZnFe_2_O_4_, and MgFe_2_O_4_, as well as their metal and carbon nanocomposites, are being developed as electrochemical biosensors for cancer detection [184,185,186]. Because of their cost-effectiveness, rapid detection, and simple operational procedure, ferrite materials are proven to be alternative electrode materials to magnetite nanomaterials and their noble metal nanocomposites. Observations from the literature survey indicate that real-time applications present the greatest challenge. In the past, all sensors were only tested in a laboratory. It is still far from commercialization or translation to end-users and needs to be verified in real-time at every stage.

## 4. Cancer Therapy

Currently, magnetic nanoparticles are used to diagnose cancer, allowing healthcare practitioners to observe cancer cells anywhere in the body. Alternatively, magnetic nanoparticles can be used in cancer therapy via chemotherapy drug delivery, stimuli-responsive drug delivery, hyperthermia, photothermal and photodynamic therapy, and magnetic nanorobots. This section provides an overview of magnetic nanomaterials and their nanocomposites that are used in the design of these therapeutic applications. The advanced applications of AI technologies in biomedicine will also be demonstrated with the use of magnetic nanoparticles in AI-integrated cancer therapy.

### 4.1. Chemotherapy Drug Delivery or Anticancer Drug Loading and Release

Chemotherapy drug delivery is a process of delivering anticancer drugs to target cancer cells while minimizing the effect on healthy tissues. Chemotherapy drugs can be delivered via a variety of methods, including intravenous injections, oral tablets, transdermal patches, and topical creams [187]. Traditional chemotherapy drugs are delivered through a systemic approach by administering intravenously and circulating them throughout the body. This systemic approach has the potential to cause significant side effects due to the drug’s non-specific targeting of healthy as well as cancerous cells [188]. To reduce the side effects and improve drug efficacy, various drug delivery systems have been developed to deliver chemotherapy drugs directly to tumors or tumor-associated tissues. Recent advancements in chemotherapy drug delivery have focused on improving the specificity of drug delivery and providing more localized targeted therapy [188]. This approach has been particularly important in the treatment of solid tumors. Drug delivery systems allow cancer drugs to be localized to the tumor site and released in a controlled manner.

Noticeably, nanostructured pharmaceutical formulations such as nanoparticles, liposomes, and polymeric micelles have demonstrated the ability to ameliorate the therapeutic effect of active pharmaceutical ingredients (APIs) [189]. Briefly, colloidal nanoparticles are engineered to carry drugs or imaging agents and can be designed to target specific sites. These nanoparticles can be loaded with drugs and released the drugs in a controlled manner at the tumor site. Yahya et al. examined the effect of lipid-based nanoparticles on drug delivery and anticancer drug release with the matrix solution system [190]. It was observed that the specific size and morphology of nanoparticles showed prolonged drug release by exhibiting higher loading capacity of various therapeutic active compounds and could be effectively employed as anti-cancerous drug delivery agents. Magnetic nanoparticles (MNPs) are emerging as promising candidates for cancer diagnosis and treatment [120]. MNPs can be used to target tumors and deliver drugs, imaging agents, and therapeutic agents directly to the site of cancer. There has been significant progress in the development of multifunctional magnetic nanostructures for cancer diagnosis and treatment in recent years [191]. These nanostructures offer several advantages over traditional methods, including their ability to target specific cancer cells and deliver therapeutic agents with high efficiency. Multifunctional magnetic nanostructures are typically composed of iron oxide or cobalt-platinum mixed-metal oxides. These MNPs can be functionalized with various biomolecules, such as antibodies or proteins, to target specific cancer cells. Once these MNPs are internalized by the cancer cells, they can be magnetically guided to the tumor site using an external magnetic field, allowing for the precise delivery of therapeutic agents. However, more clinical studies on therapeutic agents are required to provide detailed insights into these types of novel biomaterials for corneal drug delivery [192].

**Figure 8:**
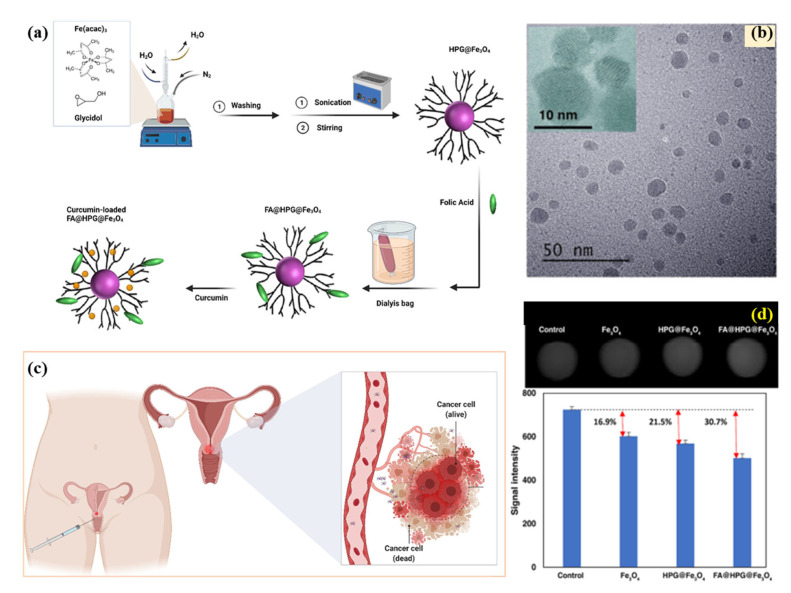
(**a**) Schematic diagram for the synthesis of HPG@Fe_3_O_4_ and FA@ HPG@Fe_3_O_4_ MHNs, (**b**) TEM image of HPG@Fe_3_O_4_ MHNs, (**c**) schematic illustration of cervical cancer treatment, and (**d**) in vitro T2-weighted MRI of HeLa cells and enhancement of signal intensity. Reprinted from Ref. [196]. Creative Commons license.

Recent studies have demonstrated that Fe_3_O_4_ NPs containing the anticancer drug doxorubicin and hydrophobic poly(ethylene glycol) (PEG) target cancer effectively [193]. The iron oxide surface is coated with the anticancer drug doxorubicin, and the organic coating is coated with folic acid. Despite the insufficient effect folic acid has on Fe_3_O_4_ surface potential, MCF-7 cancer cells readily absorb particles with moderate folic acid content on their surfaces. This research contributes to a better understanding of the structure–activity relationship in hybrid biocompatible nanosystems and opens up new opportunities for cancer theranostics. Further, doxorubicin and docetaxel can also be delivered asynchronously by magnetic hydrogels to treat patients with triple-negative breast cancer [194]. A single-drug-loaded hydrogel had significantly lower antitumor activity than a dual-drug-loaded magnetic hydrogel (DDMH). DDMH appears to be a promising multiagent co-delivery system for synergistic chemotherapy in cancer treatment, as the release of drugs is controlled by AMF triggers and has a more efficient antitumor effect than conventional cancer chemotherapy. Further, MMNPs can be used for chemotherapy/magnetic field/photothermal (chemo/MF/PTT) combination therapy, providing a potential method for loading and releasing drugs through polymeric or protein coatings [195]. According to this study based on the expression of C-X-C motif chemokine ligand 12 (CXCL12) and CXCR7 mRNA, chemotherapy/MF/PTT combined therapy exhibited the greatest reduction in breast cancer metastatic activity. Further, the Fe_3_O_4_ NPs containing folic acid (FA) and curcumin were also successfully used in the treatment of cervical cancer (FA@HPG@Fe_3_O_4_) [196]. As shown in Figure 8a, the successful preparation of Fe_3_O_4_, HPG@Fe_3_O_4_, and FA@HPG@Fe_3_O_4_ nanoparticles was demonstrated. TEM images in Figure 8b confirm that the developed FA@HPG@Fe_3_O_4_ exhibited spherical morphology with a diameter of about 10 nm and demonstrated an average size of approximately 10 nm.

The FA@HPG@Fe_3_O_4_ NPs were successfully loaded with curcumin to treat cervical cancer as schematically illustrated in Figure 8c. FA@HPG@Fe_3_O_4_ and HPG@Fe_3_O_4_ NPs had maximal drug-loading capacities of 82 and 88%, respectively. FA@HPG@Fe_3_O_4_ NPs were more readily absorbed by HeLa cells and mouse L929 fibroblasts than HPG@Fe_3_O_4_ NPs. MRI results in Figure 8d showed that FA@HPG@Fe_3_O_4_ NPs increased T2-weighted signal intensity. A poly-hydroxylated HPG@Fe_3_O_4_ nanocarrier enhanced its therapeutic potential by adding FA to the poly-hydroxylated HPG@Fe_3_O_4_ NPs. Curcumin can be loaded and released by FA@HPG@ Fe_3_O_4_ NPs to treat cancer. The MRI test results indicate that the MHNs developed in this study have the potential to help treat and diagnose cervical cancer. Drug loading and cancer therapy applications have also been conducted using ferrites of ZnFe_2_O_4_, which have a higher magnetic susceptibility than Fe_2_O_3_ or Fe_3_O_4_ [197]. As a new carrier for oil-based lipophilic drugs, oleosomes that are functionalized with recombinant proteins can be used to transport oil-based lipophilic drugs, which are ideal for the treatment of cancer. The ability of magnetic hyperthermia to increase drug release from functionalized magnetic oleosomes has shown high anticancer activity in cancer cell cultures. Moreover, the system’s efficacy was also demonstrated in an in vivo animal in that study. This makes the proposed oleosome system a very promising method for delivering drugs to breast cancer patients. Similarly, lipid-based vesicles (liposomes) can also be loaded with the desired drugs needed to cure or inactivate cancer cells [198]. A liposome is a spherical vesicle made from phospholipids, which are naturally occurring. Typically, they have a lipid bilayer delineated by an aqueous space. In addition to their multifarious composition, liposomes are non-toxic, non-immunogenic, biocompatible, and biodegradable, which makes them promising candidates for anti-cancer delivery systems [199]. Liposomes can be engineered or combined with other carrier agents to target specific cell types and also provide sustained release of the drugs [200]. Interestingly, the combination of multiple chemotherapy drugs in a nanocarrier base proves promising in an anticancer treatment by exhibiting higher drug delivery efficiency [201]. In summary, chemotherapy drug delivery systems have been designed and adopted to target specific cancerous tissues and release the drugs in a controlled and sustained manner. Additionally, the incorporation of targeting ligands or imaging agents into these systems can further enhance drug delivery and release.

### 4.2. Stimuli-Responsive Drug Delivery

Stimuli-responsive drug delivery systems are drug delivery systems that can be programmed to release drugs in response to various external stimuli [202]. This type of system is used to control the release of drugs to maximize their therapeutic efficacy, reduce their side effects, and increase their safety [203]. Some of the most commonly used stimuli include temperature, pH, light, and ultrasound. These stimuli cause a change in the material, which allows the drug to be released from the material [204]. This type of drug delivery has the potential to provide more precise and targeted drug delivery, which could lead to improved therapeutic outcomes. Research on this topic has focused on the development of materials that are responsive to the desired stimuli and are capable of releasing the drug in response to the stimuli. Researchers have explored the use of polymeric materials, such as hydrogels and nanoparticles, as well as other materials, such as liposomes, for this purpose [205,206]. Ultrasound-sensitive drug delivery systems use materials that are sensitive to sound waves, such as microbubbles, to release drugs in response to changes in sound intensity. Ultrasound can cause local high temperatures and membrane perforation, causing tumor cells to absorb drugs more readily by altering their membrane permeability [207]. Nanocarriers can release drugs by ultrasound through either the thermal or mechanical effects of cavitation or radiation forces. Physical forces exerted on non-sensitive nanocarriers cause structural destabilization, resulting in drug release [208]. This also causes blood vessels to become more permeable, allowing particles and drugs to enter and enter tumors more easily. A wide range of cancer treatments uses Fe_3_O_4_ nanoparticles as anticancer vehicles. In a recent study, Fe_3_O_4_- SiO_2_ (MPE-NDs) were successfully used as drug-loading vehicles for DOX which was delivered to the cancer cells under ultrasound stimulation [209]. Combined imaging and therapeutic functions are demonstrated in drug-loaded MPE-NDs, which provide invaluable insight into cancer treatment as well as improve ultrasound drug delivery. Under stimulation with US, DOX was successfully loaded on citrate-stabilized iron oxide nanoparticles (ML-MBs), which killed both BxPc-3 and Panc02 pancreatic cancer cells [210]. In spite of the focus of this study being pancreatic cancer, the drug delivery method could be applied to a variety of other malignancies with triggered releases of therapeutics. A magnetic delivery system triggered by ultrasound could be a promising delivery method for killing cancer cells. Meanwhile, pH-sensitive drug delivery systems release drugs at the desired pH by using magnetic materials such as polymers and liposomes. During targeted tumor therapy, pH-sensitive drug carriers are used most often since pH distinguishes normal tissues from malignant tumors in the body. In solid tumors, excessive glycolysis and poor perfusion result in acidic extracellular regions, known as the Warburg effect. The pH-sensitive drug delivery systems can deliver drugs directly to targeted cancer cells. A recent study showed that methotrexate (MTX) loaded magnetic composite materials released drugs at different pH values in an alternating magnetic field environment [211]. The release of MTX was completely observed at pH 5. It is expected that a lower pH in these tissues will result in a greater amount of drug release than in normal tissues. A pH-responsive cancer therapy using magnetic nanocubes with PMMA-functionalized hydrazone nanocarrier has been reported recently [212]. The hydrazide is decorated on PMMA to transform its hydrophobicity into hydrophilicity. Doxorubicin (DOX) was paired together with decorated hydrazide to form pH-responsive hydrazone bonds. A variety of pH and temperature values, as well as an alternating magnetic field, were used to study the in vitro release of methotrexate (MTX) into MNCPs loaded with the anticancer drug. DOX was successfully loaded and released at different pH levels, with DOX being completely released at pH 5. This system could deliver intracellular pH-responsive drugs and inhibit tumor growth remotely. Magnetic nanoparticles embedded in carbon dots@chitosan@metalorganic framework [213], taurine-conjugated mussel-inspired iron oxide nanoparticles [214], pH-labile ascorbic acid-coated magnetic nanocarriers (AMNCs) [215], mesoporous magnetic nanoparticles labeled with folic acid [216], and polymer-tethered multifunctional magnetic nanoparticles [217] have also been successfully used in pH-triggered drug release into cancer cells. Based on the results of the studies, polymer (PMMA), folic acid, and chitosan can serve as capping agents for magnetic nanomaterials to capture various anticancer drugs as well as decrease the toxicity of the drug delivery system.

### 4.3. Hyperthermia Treatment for Cancer

Hyperthermia, or thermal therapy, is a cancer treatment that uses heat to damage or destroys cancer cells and shrinks tumors [218]. Hyperthermia is a cancer treatment method in which the cancerous tissues were directly cured by heating the specific areas of a patient’s body that have a tumor. During the procedure, the patient is exposed to temperatures that are higher than normal body temperature, and the affected area is exposed to heat for several hours. As a result, the immune system responds more effectively against cancer while immune suppression inhibits the overhauling of damaged cancer cells [219]. Additionally, magnetic nanoparticles are injected directly into the tumor and then exposed to a strong magnetic field generated by an external source [16]. This field causes the particles to vibrate, creating heat which is then transferred to the tumor. The heat generated by the magnetic nanoparticles is capable of killing cancer cells without damaging healthy tissue. This type of treatment is typically administered in combination with other treatments such as chemotherapy and radiation [220]. Studies have shown that hyperthermia can be effective in treating some types of cancer, such as brain tumors and melanoma [218]. However, it is still considered experimental and more research needs to be completed to determine its effectiveness in treating other types of cancer. Another example of a multifunctional magnetic nanostructure is magnetic hyperthermia agents [16]. Magnetic nanoparticles can be heated using an alternating magnetic field, allowing clinicians to target specific regions of the body with thermal energy. This heat can be used to kill cancer cells and improve the effectiveness of chemotherapy drugs. Recent research has demonstrated the potential of magnetic nanoparticles to be used for in vivo hyperthermia cancer treatment. For instance, Jadhav et al. synthesized gadolinium (Gd)-doped manganese zinc ferrite magnetic nanoparticles (MNPs) for magnetic fluid hyperthermia (MFH) [221]. It was reported that synthesized ferrites-based MNP was non-toxic and exhibited improved structural, colloidal, and magnetic properties, and heating performance. The developed MNPs deactivate A549 cancer cells at a rate of up to 61% within 1 h of in vitro MFH treatment time, and hence, could be effectively employed for cancer cell treatment [221]. 

**Figure 9:**
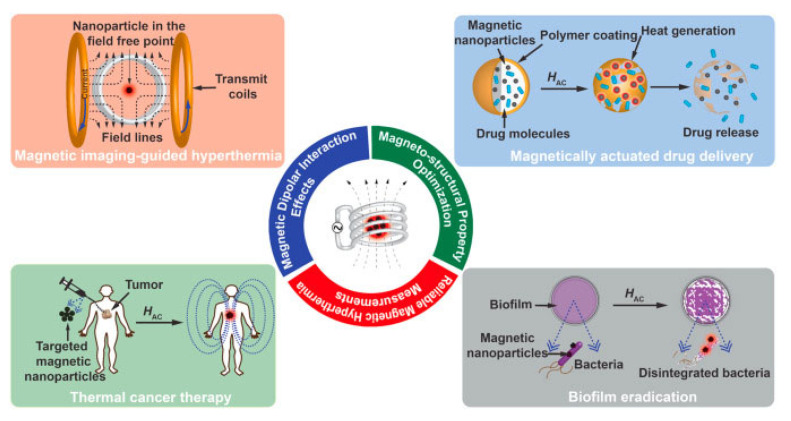
Schematic illustration of magnetic nanoparticle-mediated hyperthermia for cancer therapy. Reprinted from Ref. [225]. Copyright 2016 Elsevier.

Another study reported the use of oleic-acid-stabilized iron-oxide magnetic nanoparticles for curing transplanted Walker 256 carcinoma tumors via the magnetic hyperthermia technique. The results revealed that MNP hyperthermia survived six out of a total of seven animals suffering from cancer, whereas none of the animals survived in a control (untreated) group [222]. Hence, MNPs could be effectively employed to cure cancer cells through the hyperthermia technique. In addition, magnetic nanoparticles have been tested for hyperthermia treatment of prostate cancer cells [223,224]. The results revealed that exposure to alternating magnetic fields, combined with magnetic nanoparticles, led to increased cell death in the cancer cells. Overall, the research suggests that magnetic nanoparticles may be a promising tool for use in the hyperthermia treatment of cancer. Further research is needed to fully understand the potential of magnetic nanoparticles for use in hyperthermia treatments to cure various kinds of cancer tissues. Figure 9 presented the schematic illustration of magnetic nanoparticle-mediated hyperthermia for overall cancer therapy applications.

**Figure 10:**
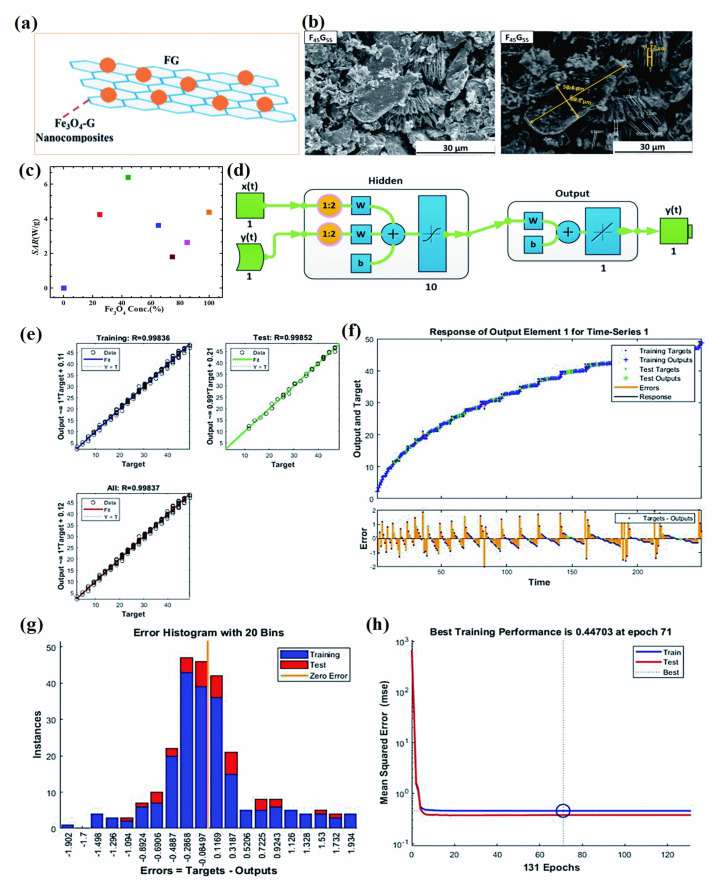
(**a**) Schematic illustration of Fe_3_O_4_-G, (**b**) SEM images of F_45_G_55_, (**c**) SAR values of Fe_3_O_4_-G with different compositions, (**d**) neural network architecture, (**e**) the output and target correlation for sample F_45_G_55_, (**f**) time series response curves with time instances on the *x*-axis and predicted versus observed values on the *y*-axis, (**g**) error histogram with 20 bins, and (**h**) performance plot of MSE versus epoch count. Reprinted from Ref. [226]. Creative Commons license.

Furthermore, MHT applications using magnetic nanoparticles can be predicted and optimized with artificial neural networks (ANN). This ANN system used several learning algorithms to assess the performance of hyperthermia. Recently, Fe_3_O_4_ nanoparticles (FeNPs) have been successfully developed for MHT applications, and multiple ANNs have been developed to assess the high MHT accuracy, including BFGS Quasi-Newton (ANN-BFG), Levenberg-Marquardt (ANN-LM), and Bayesian Regularization (ANN-BR). An ANN-BFG learning algorithm is effective for multilayered networks [21]. An ANN-LM was used due to its faster convergence rate, whereas an ANN-BR prevented the network from overtraining and overfitting. This study investigated the holistic effects of particle concentration, AMF P, and exposure time on localized TH using an ANN approach. The ANN-BFG speed was optimized using particle swarm optimization (PSO). The hybrid ANN-PSO model predicted localized temperature with excellent accuracy (42–47 ^◦^C range), convergence (less than 7), and precise optimization under hyperthermia conditions, such as particle concentration, AMF *P*, and exposure time. The optimal composition of graphene-Fe_3_O_4_ nanohybrids used in magnetic hyperthermia was also estimated with the help of ANN, as shown in Figure 10a–h. A study showed that Fe_3_O_4_ NPs graphene nanosheets (G-Fe_3_O_4_) have a uniform dispersion, high biocompatibility, and high thermal conductivity, making them ideal candidates for magnetic hyperthermia [226]. For studying their hyperthermia performance, the G-Fe_3_O_4_ were exposed to an alternating current magnetic field at a frequency of 633 kHz and a strength of 9.1 mT. An ANN model was used to investigate localized antitumor effects. An ANN model was used to investigate localized antitumor effects. Based on the neural net time-series model, the best nanohybrid composition was almost 100% accurate. The NARX models used in this study included external inputs for each component of the model. The accuracy of predicted results has been assessed by mean square error (MSE). F_45_G_55_ is a model containing 45% magnetite and 55% graphene that achieved optimal results after 71 epochs in the training phase. It was found that F_45_G_55_ nanohybrids had the highest mean squared error for hyperthermia applications with low doses and a high specific absorption rate (SAR). These studies concluded that ANN models can be successfully used in the design and development of magnetic nanoparticles for MHT applications. Meanwhile, ANN models have been used to evaluate the cytotoxicity of nanoparticles as a function of their size [227]. Particle size, concentration, incubation time, and surface charge of nanoparticles were selected as inputs for the ANN model, and percentage cell viability (%CV) as output. Magnetic nanoparticles with greater hydrodynamic sizes have a lower chance of penetrating cells; thus, they have a higher %CV. In this model, the zeta potential of nanoparticles was examined under different laboratory conditions. It was concluded that HEK293-T cells adhered better to NPs with higher PZP. However, a robust algorithm requires as much information as possible to work effectively.

### 4.4. Photothermal and Photodynamic Therapy

Photothermal and photodynamic therapy (PTT/PDT) utilizes energy from light to treat cancer cells. PTT/PDT uses light energy to generate heat or light-activated drugs to kill cancer cells. PTT/PDT uses a photosensitizer, which is a light-activated drug that is absorbed by cancer cells and then activated by light energy to generate heat and destroy cancer cells [228]. In PTT, a laser is used to generate heat that kills cancer cells. PTT/PDT is effective in treating certain types of cancer, including head and neck cancer, bladder cancer, and some forms of skin cancer [229]. Magnetic nanoparticles (MNPs) have also been used in both photothermal and photodynamic therapies. In photothermal therapy, photosynthesizing agents such as magnetic nanoparticles (MNPs) are injected into the body and absorbed by the cancer cells. The agent is then activated by a specific wavelength of light, which generates heat to exterminate the cancer tissues. This therapy is usually used in combination with other cancer treatments, such as chemotherapy or radiation therapy.

**Figure 11:**
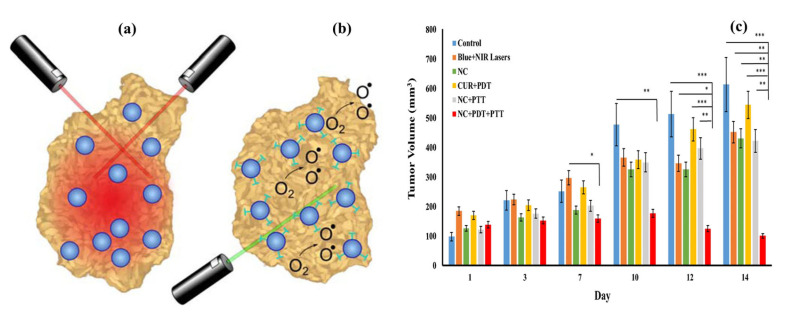
Tumor ablation therapies with iron oxide NPs. (**a**) In photothermal ablation, light absorbed by NPs is converted to thermal energy causing cell death in the vicinity. (**b**) For photodynamic therapy, photosensitizing agents attached to NPs are activated by an external light source to create singlet oxygen species that are cytotoxic to cells. Reprinted from Ref. [231]. Creative Commons license. (**c**) Average tumor volume for different treatment groups using Blue + NIR lasers, CUR + PDT, NC + PTT, and NC + PDT + PTT employing over 2 weeks [232]. Reprinted from Ref. [232]. Copyright 2020 Springer Nature. * *p* < 0.05, ** *p* < 0.01, and *** *p* < 0.001.

Figure 11a,b presents the tumor ablation therapies with iron oxide NPs via photothermal ablation and photodynamic therapy, respectively. In photodynamic therapy, MNPs are injected into the body and then exposed to light, resulting in a chemical reaction that generates a toxin that kills the cancer cells [230,231]. The agent is then activated by a specific wavelength of light, which generates oxygen radicals and targets the tumor site. This therapy is often used to treat cancer of the skin, bladder, and esophagus, as well as some types of leukemia. Ashkbar et al. investigated the effect of magnetic nanocomposite (NC) for breast cancer in vivo treatment by adopting dual PDT and PTT approaches [232]. The results revealed that tumor volume showed a maximum of 94% reduction for NC+PDT+PTT compared to other treatment strategies as shown in Figure 11c. In summary, both photothermal and photodynamic therapies have the potential to be less toxic and more targeted than traditional chemotherapy, as they specifically target cancer cells and do not affect healthy cells. However, these therapies are still relatively new and more research is needed to fully understand their effectiveness for different tumor types using novel and economic magnetic nanomaterials.

### 4.5. Magnetic Nanorobots

Magnetic nanorobots are a type of nanorobot that are designed to deliver anticancer drugs to specific locations within the body. These nanorobots are typically made of a magnetic material, such as iron oxide, are coated with a protective layer of polymers or other biocompatible materials, and can be guided to a specific location within the body using an external magnetic field [233]. They can navigate through the body using external magnetic fields, allowing them to be directed to specific areas of the body with high precision. One of the key advantages of using magnetic nanorobots for drug delivery is their ability to target specific areas of the body with high precision and their ability to navigate through complex environments, such as vasculature or tumor tissue [234]. This allows them to deliver the drugs directly to the cancerous cells, reducing the potential for side effects and improving the effectiveness of the treatment. Once they reach their destination, they can release the anticancer drugs, either through passive or active drug release mechanisms. Additionally, magnetic nanorobots can carry a larger payload of drugs than traditional drug delivery methods, allowing for more effective treatment [235]. They are also able to release the drugs in a controlled manner, ensuring that the optimal dose is delivered to the targeted cells. Overall, magnetic nanorobots have the potential to revolutionize the way that anticancer drugs are delivered, offering a more precise and effective approach to cancer treatment.

**Figure 12:**
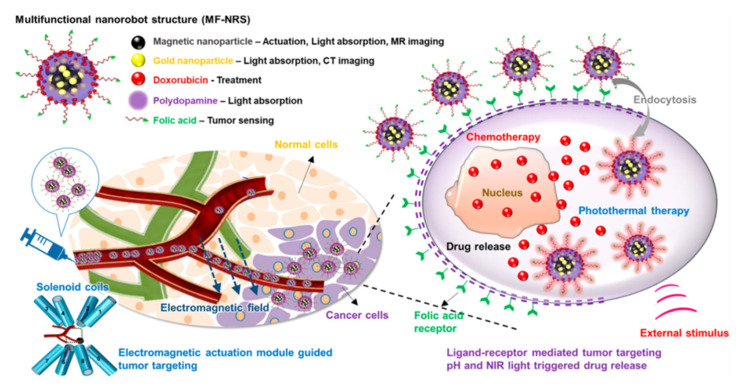
Multifunctional magnetic nanorobot structure diagram for cancer therapy [238]. Reprinted from Ref. [238]. Copyright 2019 ACS.

There have been several studies that have explored the use of magnetic nanorobots for anticancer drug delivery. For example, a study demonstrated that magnetic nanorobots could be used to deliver doxorubicin, a commonly used chemotherapy drug, to breast cancer cells in vitro [236]. The nanorobots were able to significantly reduce the number of cancer cells, while also reducing the toxicity of the drug to normal cells. Other studies have also shown promising results for the use of magnetic nanorobots in the delivery of anticancer drugs. Magnetic nanorobots have been used to deliver cisplatin, another chemotherapy drug, to lung cancer cells in vitro [237]. The developed nanorobots were able to significantly reduce the number of cancer cells and increase the survival rate of the mice. Recent developments offer multifunctional nanorobot systems that can load chemotherapy drugs precisely, trigger safe drug releases with light, perform photothermal therapy with light, and provide enhanced magnetic resonance imaging [238]. Photothermal therapy and chemotherapy are synergistic antitumor effects in vitro, and the nanorobot system exhibits outstanding tumor targeting efficiency both in vitro and in vivo, as schematically depicted in Figure 12. Nanorobots kill more than 84.5% of Hep3b cells within 24 h using photothermal therapy and chemotherapy. In vitro and in vivo, the system kills more than 63.7% of Hep3b cells. In addition to being an imaging contrast agent, MF-NRS demonstrated its potential as a tumor-size analyzer, after which its therapeutic effects can be evaluated. Based on the findings of this study, nanorobot systems for biomedical applications such as cancer treatment and active drug delivery systems should be investigated further. The results demonstrated that magnetic nanorobots can be used for the delivery of anticancer drugs. Further research is needed to fully explore their potential benefits and limitations and to optimize their design and drug delivery mechanisms.

The reported results ensured the applicability of magnetic nanorobots for anticancer drug delivery applications. However, further research is needed to deeply explore the potential benefits and limitations of this technology, as well as to optimize its design and drug delivery mechanisms.

### 4.6. Limitations of Using Magnetic Nanostructures in Cancer Therapy

The use of magnetic nanomaterials (MNPs) in cancer treatment has advanced rapidly, but some issues are still unresolved. The toxicity of MNPs is still a major concern. The small size of MNPs also makes them capable of penetrating physiological barriers, which can be harmful to health. Specifically, MNPs disrupt cell viability, leading to membrane leaks, and impair metabolism, and proliferation [120]. The acidic local environment of cancer cells reduces MNPs which produce toxic elements and free radicals during targeted delivery [239]. The release of free radicals from MNPs may damage cellular membranes, organelles, and DNA. Several factors influence MNPs toxicity, such as dosage, size, biodegradability, solubility, etc. Consequently, MNPs need to be assessed accurately for their toxicity. Recent studies have shown that the surface of MNPs can be functionalized in a variety of ways, which reduces their toxicity and improves their stability. MNPs surfaces modified with functional groups enable derivatization and high solubility in a wide range of solvents. To reduce the health risks associated with MNPs, the following suggestions for reducing their toxicity may be worth considering for cancer diagnosis and cancer therapy applications [240,241,242].

(1) Several factors determine the toxicity of MNPs, including their administration method, their biodegradability, stability, and their surface chemistry. Biocompatible polymeric materials and co-polymers can be used to functionalize MNPs. Especially, incorporating biocompatible polymers into functionalized MNPs surfaces may enhance particle stability, dispersibility, and biocompatibility.
(2) Ni, Co-ferrite based magnetic materials are widely used in several biomedical applications, however, it shows significant toxic effects during cancer diagnosis and cancer therapy applications. Particularly, Co-ferrite materials have a high coercivity and magnetization, making them potential candidates for hyperthermia applications. The toxicity of this material is higher than that of iron-oxide-based superparamagnetic materials. Carbon or graphite coatings will prevent the toxicity of Co-ferrite based materials and facilitate targeted delivery, whereas polymer functionalization with carbon-coated MNPs will enhance biocompatibility and drug-loading ability.
(3) The coating of noble metals, such as Au, Ag, or Pd, may reduce the toxicity of MNPs, which have longer circulation lifecycles, and increase therapeutic drug availability. Further, Au@MNPs have photomagnetic properties that may help advance photothermal therapy and cancer diagnosis in the future.
(4) Magnetic hybrid nanostructures conjugated with proteins, DNA, and other biomolecules are promising tools for improving cancer diagnosis and therapy while also reducing their toxicity.
(5) Artificial intelligence (AI) will enable the design, composition, functionalization, dosage optimization, loading, and assessment of the toxicity of MNPs and their hybrids.

It is imperative to conduct long-term studies since the toxic effects of MNPs may not be evident for years after prolonged exposure due to the non-biodegradable nature of many commercial MNPs. There have been few long-term toxicity studies conducted to date, and a more accurate recreation of the tumor microenvironment is often overlooked in in vitro assays. In spite of this, it is important to understand how MNPs affect human health and the environment, which is mostly dependent on AI technology. 

### 4.7. Administrative Strategies for Nanoparticles

There are several strategies for administering magnetic nanoparticles in anticancer drug delivery, including intravenous injection, intratumoral injection, and targeted delivery to specific organs or tissues [243]. Briefly, intravenous injection is the most common method of administering magnetic nanoparticles. This involves injecting the nanoparticles into the bloodstream, where they can be directed to cancerous tumors via the use of an external magnetic field. This approach is effective in targeting and delivering drugs to liver and lung tumors. Additionally, intratumoral injection involves injecting the nanoparticles directly into the tumor site [244]. This method has the advantage of delivering a higher concentration of the drug directly to the tumor, increasing the likelihood of tumor cell death. However, this method may be less effective in reaching tumors in deeper tissues or those that have spread to other parts of the body. Another strategy adopted for cancer treatment is targeted delivery which utilizes specific targeting agents, such as antibodies or peptides, to direct the nanoparticles to specific organs or tissues [245]. This method has the potential to improve the specificity and efficiency of drug delivery, reducing the risk of side effects. However, the development and use of targeting agents can be complex and costly. Furthermore, there is ongoing research into the use of magnetic nanoparticles for hyperthermia using external beam radiation [246]. This method utilizes magnetic nanoparticles in the presence of external radiation beams to enhance the effectiveness of the radiation treatment by increasing the absorption of the radiation by the targeted cancerous tumor sites. This approach has shown promising results in early studies, but further research is needed to fully evaluate its effectiveness and safety. In addition to their use in cancer treatment, multifunctional magnetic nanostructures are also being explored for use in cancer diagnosis [247]. For example, MNPs can be functionalized with biomarkers or contrast agents and used in magnetic resonance imaging (MRI) to detect and monitor cancer progression. Overall, multifunctional magnetic nanostructures showed great potential for improving cancer diagnosis and treatment. A summary of the work on magnetic hybrid nanostructures for cancer diagnosis and therapy is presented in Table 1. Further research is needed to optimize their design and test their clinical use.

### 4.8. Applications of Artificial Intelligence for Cancer Treatment and Diagnosis

Artificial intelligence (AI) has the potential to revolutionize the field of cancer diagnosis and anticancer drug delivery systems [248]. By using machine learning algorithms and data analysis, AI can diagnose cancer at an early stage and/or helps in treating cancer by optimizing the drug delivery to specific tumor sites, reducing side effects, and improving treatment outcomes. Briefly, AI has been implied to analyze imaging data and predict the likelihood of cancer recurrence after surgery [249]. This information can help doctors tailor treatment plans and increase the likelihood of a successful outcome. Additionally, AI has also been used to identify patients at risk of developing cancer by analyzing electronic health records. This early detection can help prevent the disease or allow for early treatment, improving patient outcomes [250]. Besides cancer diagnosis, artificial intelligence (AI) can be used in various ways to improve the delivery of anticancer drugs to patients. Some potential applications include predictive modeling, targeted drug delivery, dosing optimization, real-time monitoring, and adverse event prediction. Concisely, AI algorithms can be used to predict the likelihood of a patient responding to a particular drug or treatment regimen. It will improve the accuracy and effectiveness of the targeted drug delivery and can help doctors tailor treatment plans more effectively by minimizing the likelihood of any side effects. AI algorithms can also be used to optimize drug dosing for individual patients based on factors such as weight, age, and another medical history, and hence, reduce the risks of overdose or underdose and result in improved treatment outcomes [251]. Recently, AI algorithms have also been adopted to continuously monitor the response of a patient to a particular drug or treatment regimen in real time. This can help doctors make adjustments to treatment plans as needed to ensure the best possible outcomes. Importantly, AI algorithms can be used to identify patterns or indicators that may predict the possibility of an adverse event occurring during treatment so the doctors take preventative measures to minimize the risk of such events. Recently, liquid biopsies utilizing circulating tumor DNA or cell-free DNA (cfDNA) are emerging as ways to detect cancer early via AI. Cohen et al. developed CancerSEEK to detect and predict eight cancer types early using ctDNA via DL models [252]. In the future, as liquid biopsy data acquisition increases, DL models will allow for the combination of multiple data types to enhance early cancer detection, eliminating the need to manually select and curate discriminatory features [253,254].

MNPs (anti-cancer agents) also have the potential to be used in combination with artificial intelligence (AI) to create novel diagnostic and treatment strategies [255,256,257]. For instance, AI-enabled nanoparticle synthesis platforms and nanoparticle delivery systems (AI-assisted algorithms) have been adopted to optimize the synthesis and delivery of magnetic nanoparticles for anticancer drug delivery, respectively. It uses machine learning, deep learning, and computer vision techniques to predict the optimal parameters for nanoparticle synthesis that accurately target the nanoparticles to the desired cells [258,259]. Furthermore, AI-assisted algorithms have also been employed to track the magnetic nanoparticles and monitor the toxicity of the drug during delivery to provide insights into cancer progression and identify potential therapeutic strategies [260]. It uses natural language processing, deep learning, and computer vision techniques to accurately detect the nanoparticles at the specified locations (tumor sites) and assess the levels of toxicity of the drug, respectively. One study found that AI-based drug delivery systems resulted in a significant reduction in tumor growth and improved survival rates in mice models [261]. Another study found that an AI-based system was able to accurately predict which patients would respond positively to a specific drug regimen, allowing for personalized treatment approaches. However, more research is needed to fully understand the potential of AI in anticancer drug delivery and to address potential ethical concerns.

**Figure 13:**
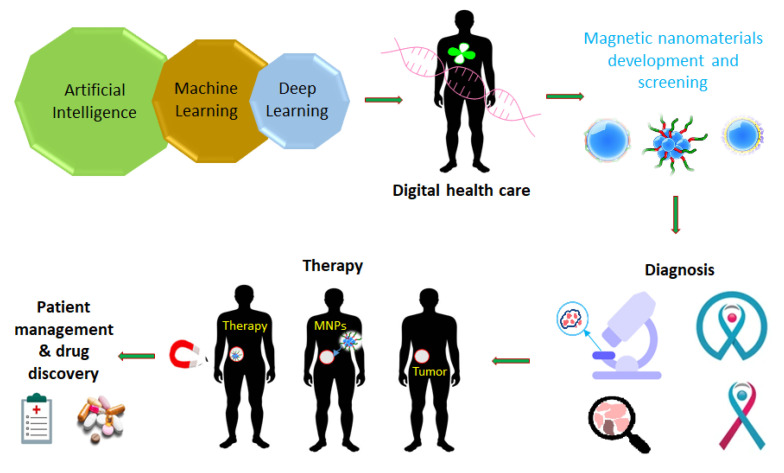
Schematic visualization of artificial intelligence in the precise diagnosis and treatment of liver cancer.

Additionally, DeepCare, HyperView, and ThermAI platforms have been designed to help clinicians optimize and automate the planning and analysis of hyperthermia cancer treatments [262,263]. These algorithms predict the best heating patterns to maximize the effectiveness of hyperthermia therapy while minimizing collateral damage to healthy tissues, whereas photothermal or photodynamic cancer treatment has been visually monitored by AI-based image analysis software to examine images of the treatment area and help determine the optimal location and intensity of the light during light-based cancer treatment techniques. Figure 13 schematically presented the applications of AI technology in the diagnosis and treatment of cancer. Briefly, six application scenarios include virtual assistants, medical imaging diagnosis, adjuvant therapy, risk screening/treatment or prognosis evaluation, drug development/testing, and postoperative rehabilitation management. In summary, the use of AI in anticancer drug delivery shows promising results to improve patient outcomes and reduce healthcare costs by optimizing treatment plans and increasing the effectiveness of drugs.

## 5. Concluding Remarks and Perspective

Recent research has focused on developing multifunctional magnetic hybrid nanostructures (MHNs) capable of imaging and delivering cancer-targeted therapies to patients. MHNs have been extensively used in recent years as T1 and T2-weighted MRI contrast agents, drug delivery devices, magnetic sensors, and hyperthermia-generating probes. For cancer diagnosis, MHNs larger than 10 nm are effective as T2 MRI contrast agents, whereas MHNs smaller than 5 nm are effective as T1 MRI contrast agents. In both T1 and T2 MRI contrast, MHNs prove effective in terms of both their size and surface functionalization as long as both are optimized. Furthermore, MHNs may be used to manufacture microchips capable of detecting cancer earlier using an early detection system. In the future, a low-cost magnetic microchip system may be able to detect cancer earlier. A GMR nanosensor offers the advantages of both technologies—sensitivity, and versatility in addition to a low price and quick test time for the diagnosis of cancers. However, biosensors based on GMR require specialized magnetic wafers, making their implementation more challenging than sensors based on magnetic immune systems or electrochemical sensors. GMR biosensor arrays combined with CMOS electronics enable high-resolution brain imaging and multiplexed bioassays. Additionally, the use of ferrite-based electrochemical sensors would be an effective method for developing portable devices for the detection of cancer since they are highly sensitive. Compared to magnetite nanomaterials and their noble metal nanocomposites, ferrite materials offer the advantages of lower costs, rapid detection, and a simple operational procedure. Through the combination of magnetics and electrochemistry, cancer biomarkers can be detected in circulating blood. Commercialization and translation to end users are still far off, and each stage of the process must be verified in real time. Moreover, several other issues need to be carefully checked to ensure that electrodes are stable, repeatable, reproducible, and repeatable. A biosensing application relies on MHNs with advanced surface functionality. Among the key developments are the accurate control of MNPs as well as their uniform size distribution, crystal structure, and shape. The magnetic properties have been standardized by recent developments in preparation procedures, and this allows for the magnetic properties to be stabilized and thus used in very sensitive biosensors for cancer detection. 

In cancer therapy, magnetic nanoparticles such as Fe_3_O_4_, α-Fe_2_O_3_, ferrites, core-shell Au@Fe_3_O_4_, fluorescent CdS@Fe_3_O_4_, and carbon quantum dots@magnetic nanomaterials were successfully used in drug delivery vehicles, magnetic hyperthermia, stimuli-responsive drug delivery, photothermal and photodynamic therapy, and magnetic nanorobots. The development of ultra-small Fe_3_O_4_ nanoparticles grafted with fluorescent labels has been demonstrated to be useful for the T1-weighted MRI diagnosis system and treatment of cancer in several studies. Various magnetic nanomaterials have different shapes and sizes that may influence their magnetic properties, which can have a considerable impact on hyperthermia cancer treatment. Moreover, using fluorescent labeling and polymer functionalization of ultra-small Fe_3_O_4_ nanoparticles as a drug delivery system will be proposed for multimodal cancer imaging and cancer therapy. The diagnosis and treatment of various kinds of cancer have progressed significantly, but there are still several important challenges to overcome. The optimal design of clinically relevant MHNs should include factors such as stability, tracking, the release of drug components only at the target sites, and minimal risk. The particle sizes and size distributions must be reproducible, and there must be cost-effective synthetic routes that can yield large quantities of chemicals. Magnetic nanostructures have made considerable progress in a relatively short time, indicating that clinical applications are inevitable. Moreover, artificial intelligence (AI) in anticancer drug delivery has shown promising results in improving patient outcomes and reducing healthcare costs through optimizing treatment plans.

## Figures and Tables

**Table 1:** The studies of the application of MNPs in cancer diagnosis and treatment.

Nanoparticle Type	Targeting Agent	Target	Status	Applications	Treatment Type	Results	Ref.
DOX@ES-MION@RGD_2_@ mPEG	3.6 nm ES- MIONs for T1-weighted	cancer cells and tumor-bearing mice	clinical	tumor	MRI and chemotherapy	3.6 nm is the best particle size for ES-MIONs to be utilized as a T1-weighted MR contrast agent.	[18]
MTMNPs (polyethyleneimine coated Fe_3_O_4_)	EPPT peptide (Glu-Pro-Pro-Thr)	overexpressed MUC-1 receptors	in vitro	breast cancer	electrophoresis	MTMNPs improved the efficiency of gene delivery in 10% serum medium by more than 2.98-fold.	[34]
hierarchically nanostructured magnetic hollow spheres	ibuprofen	anti-inflammatory drug	-	simulated body fluid (SBF)	drug delivery	The developed material exhibited higher drug loading and release properties.	[50]
CuFe_2_O_4_ MNPs	-	-	-	cancer cells	hyperthermia	Ferrites-based MNPs showed suitability for mild hyperthermia applications at an optimum nanocomposite concentration of 1 mg/mL and a frequency of 300 MHz.	[46]
temperature-responsive magnetite/polymer nanoparticles	-	-	-	temperature-responsive volume-transition property	drug release	By increasing the temperature from 20 to 35C, hydrodynamic diameter underwent a sharp decrease from 45 to 25 nm, respectively.	[57]
star-like block copolymer with MGNPs	quercetin	cancer treatment and controlled-release	in vitro	-	drug delivery and release	Cytotoxicity showed that quercetin-loaded micelles were 69% and 44%, after 24 and 48 h.	[58]
copolymer with SPIONs	doxorubicin	HeLa and CT26 cells	in vitro	drug release	combination therapy with hyperthermia and chemotherapy	The targeted nanocarrier exhibited higher cancer-combined chemotherapy and hyperthermia.	[59]
SN-38/USPIO-loaded siRNA-PEG mixed micelleplexe	cationic PDMA-block-poly(ε-caprolactone) (PDMA-b-PCL) micelles	tumors	in vivo	tumor treatment	combine gene silencing and chemotherapy	The developed theranostic micellar drug and gene delivery system served as negative MRI contrast agents; not only serving for diagnosis but also used for tracking the therapeutic outcomes.	[60]
polymers-Ag NPs	curcumin			wound healing	pH-based drug loading and release	Ag-NPs resulted in an increased loading from 21 to 56%.	[64]
Mg-ferrites NPs	doxorubicin (DOX)	human embryonic kidney (HEK293), colorectal adenocarcinoma (Caco-2), and breast adenocarcinoma (SKBR-3) cell lines	in vitro	cancer treatment	pH-responsive drug delivery	Chitosan-based ferrites NPs showed the highest DOX encapsulation of 85%.	[65]
IONPs	calcium hydroxide Ca(OH)_2_, Taxotere (TXT)	sortilin (SORT-1, a human IgG1 monoclonal antibody)	in vitro, ex vivo and in vivo	caov-4 ovarian cancerous cells	targeted drug delivery	Ca(OH)_2_@Fe_3_O_4_/PVA/Au-SORT nanotherapeutics inhibit tumor growth by 78 % and is even effective on aged tumors.	[66]
carbon-coated FeCo	-	mice tumor	in vivo	tumor ablation in mice	cancer imaging and hyperthermia therapy	The prepared NPs were used for tumor ablation in mice and were good for photoacoustic imaging.	[72]
MCNPs	CD44 monoclonal antibodies,	breast cancer cell	in vitro	cancer cells diagnosis	fluorescence/MRI dual imaging,	Developed MCNPs-CD44 probe distinguished 4T1 breast cancer cells from normal cells and detected as low as a few hundred cancer cells.	[69]
MG-PB	DOX	-	in vitro	controlled drug release	pH-responsive drug delivery	~65% of DOX release in pH 5.0, 40 °C using MG-PB.	[70]
FA-GdN@CQDs-MWCNTs	DOX	-	In vivo	dual-modal fluorescence (FL)/magnetic resonance (MR) imaging	chemo-photothermal synergistic therapy	The developed materials could be used for simultaneous FL/MR imaging, PTT therapy, and drug delivery.	[78]
mMWNTs-GEM and GEM-mACs	-	cancer with lymph node involvement	in vitro and in vivo	drug delivery	intra-lymphatic delivery of chemotherapeutics	Functionalized MWCNTs highlight the clinical potential for future cancer metastasis treatment.	[79]
Fe-MWCNTs-Gd	human serum albumin	-	-	MRI imaging and hyperthermia treatment	cancer therapy	Dual-functioning MRI imaging and magnetic hyperthermia structures for cancer therapy	[80]
FVIOs-GO	calreticulin	4T1 breast cancer cell surface	in vitro and in vivo	breast cancer	magneto thermodynamic therapy	Developed material exhibiting antitumor capabilities and could be used for future cancer magnetotherapies.	[81]
AuNRs-Alb-NPs	-	glioblastoma N2a tumor-bearing mice	in vivo and in vitro	targeted drug delivery	photothermal therapy	Albumin NPs enhanced tumor targeting and resulted in much better tumor ablation.	[89]
AuNPs-New Sor	-	EGFR and VEFR-2	in vitro and in vivo	tumor treatment	suppressing tumor migration, and angiogenesis	AuNPs-New Sor may attenuate tumor development and angiogenesis through the downregulation of EGFR and VEGFR-2	[94]
AuNS	DOX	mice bearing human Bel-7402 hepatoma	in vivo	targeted drug delivery	photothermal-chemotherapy	Combined photothermal and chemotherapy treatment through Au nanoshells is effective for killing cancer cells and targeting drugs.	[95]
quantum dots with Fe_3_O_4_-filled carbon nanotubes (CNTs)	DOX	HeLa cells	in vitro	drug delivery	simultaneous cancer-targeted optical imaging and magnetically guided drug deliver	The developed nanocarrier exhibits multifunctional features such as drug loading, optical imaging, and magnetically guided drug delivery.	[106]
Fe_3_O_4_@SiO_2_@al/ CQDs	DOX	-	in vitro	drug delivery and bio-imaging	pH-responsive drug delivery	Multifunctional magneto-fluorescent NPs exhibited a higher rate of drug release in a simulated tumor environment compared to normal tissues.	[111]
MGC-FU	5-fluorouracil	A549 cancer cells	in vitro	drug delivery and magnetic resonance/ fluorescence imaging	bimodal MRI/FI and pH-responsive drug delivery	Nanocarrier exhibits 90% of drug loading capacity and pH-dependent release.	[112]
SPIONs	siRNA	breast cancer cells	in vitro	targeted drug delivery and release	magnetically driven anti-cancer drug loading	Nanoparticles efficiently delivered siRNAs molecules without cytotoxicity.	[117]
VNFG	-	murine breast cancer cells (4T1)	in vivo and in vitro	theranostic tumor treatment	MRI-guided magnetic thermal cancer ablation	VNFG exhibited excellent magnetic thermal properties (984.26 W/g).	[125]
Fe_3_O_4_@Au-C225	-	human glioma in nude mice (UT51 cells)	in vitro and in vivo	cancer cells detection	MRI imaging	The developed MNPs adsorbed the cancer cells and could be used to trace the glioma cell line by MRI.	[127]
uIONPs	-	orthotopic murine model of glioblastoma		theranostics of brain tumor	imaging and targeting drug delivery	Ultrafine MNPs showed six-fold higher performance for molecular imaging and treatment of brain tumors.	[132]
Magnetic CuFeSe_2_ Ternary nanocrystals	-	heart, liver, spleen, lung, and kidney of tumor-bearing mice	-	photothermal ablation of tumor cells	multimodal-imaging-guided photothermal therapy of cancer	CuFeSe_2_ nanocrystals showed high photothermal conversion efficiency (82%)	[146]
HFCNPs	DOX	heart, liver, spleen, lung, and kidney of tumor-bearing mice	-	imaging-guided combined chemo/ photothermal therapy	pH/IR-based drug delivery and imaging	HFCNPs showed a high DOX loading capacity of 61.2%.	[148]
NiFe_2_O_4_ NTs		lipoprtein receptor (LSR)		ovarian cancer marker	imaging and detection	NiFe_2_O_4_ NTs provided a new thought to constructing dual-mode immunosensor.	[179]
MNCPs	methotrexate	MCF-7 human breast cancer cells	in vitro	breast cancer cells treatment	pH-responsive drug release and hyperthermia therapy	MNCPs/MTX showed 17% higher antiproliferative activity relative to that of free MTX	[210]

## Data Availability

Data will be available on request.
